# Characterization of Induced High Yielding Cowpea Mutant Lines Using Physiological, Biochemical and Molecular Markers

**DOI:** 10.1038/s41598-020-60601-6

**Published:** 2020-02-28

**Authors:** Aamir Raina, Rafiul Amin Laskar, Younas Rasheed Tantray, Shahnawaz Khursheed, Mohd. Rafiq Wani, Samiullah Khan

**Affiliations:** 1 Mutation Breeding Laboratory, Department of Botany, Aligarh Muslim University, Aligarh, India; 2 Botany Section, Women’s College, Aligarh Muslim University, Aligarh, India; 3 Department of Botany, Punjabi University, Patiala, India; 4 Department of Botany, Abdul Ahad Azad Memorial Government Degree College, Srinagar, Jammu and Kashmir India

**Keywords:** Plant genetics, Plant breeding

## Abstract

Cowpea, *Vigna unguiculata* (L.) Walp. is an important grain legume grown in the dry agro-ecologies of the tropics with considerably low yield due to lack of improved varieties, aggravated by prevalent narrow genetic base. Thus, induced mutagenesis was employed using sodium azide and gamma rays to increase genetic variability in cowpea genotypes that resulted in isolation of eleven high yielding mutant lines at the M_4_ generation from the genetic background of cowpea varieties Gomati VU-89 and Pusa-578. In order to analyze the induced genetic divergence among the mutant lines and parent genotypes, biochemical and molecular characterization was carried out with sodium dodecyl sulfate-polyacrylamide gel electrophoresis (SDS-PAGE), simple sequence repeat (SSR) and CAAT box derived polymorphism (CBDP) markers. Activity of nitrate reductase (NR) and content of chlorophyll, carotenoid, protein and mineral were found to be significantly high in the selected mutant lines compared to their respective parent genotypes. SDS-PAGE profile of seed proteins generated 54 and 28 polymorphic bands and a total polymorphism of 62.06 and 41.17% in Gomati VU-89 and Pusa-578, respectively. SSR primers amplified a total of 16 and 24 polymorphic bands with an average polymorphism of 20.69 and 50.74% in Gomati VU-89 and Pusa-578, respectively. CBDP markers, used for the first time in mutagenized population, generated 175 bands with 77 bands being polymorphic in Gomati VU-89 and 121 bands with 59 bands being polymorphic in Pusa-578. Physiological, biochemical and molecular profiling of the selected promising mutants lines showed that Gomati VU-89-G and Pusa-578-C are genetically most diverged high yielding genotypes with significant increase in protein and micronutrient content, therefore, could be recommended for further research considerations. Thus, the favorable combination of genes induced in the novel cowpea mutants selected through the present study are valuable to correspond farmers requirements for new improved cultivars (direct or hybrids).

## Introduction

Pulses are the chief components of the agricultural system, effectively boosting food and nutrition, revenue and environment across the globe and hence assumed ideal for acquiring food security in the developing world including India. Even though India is the leading producer of pulses with 26% of the total world production harvested at about 35% of the total world area, yet a prominent yield gap of 18% exists and one of the leading importer nation^1^. Cowpea [*Vigna unguiculata* (L.) Walp.] is a vital leguminous crop growing in the semi-arid tropics of Asia, Africa, Southern Europe, Southern United States, and Central and South America^2^. Cowpea leaves, green pods, and grains are excellent dietary source of protein, thus used as food and feed for healthy growth of both humans and livestock^3^. Cowpea is reportedly considered to be first originated and domesticated in Southern Africa, and later spread to east and West Africa and Asia^4^. Worldwide about 6.5 million metric tons of cowpea are produced annually on about 14.5 million hectares^5^. Cowpea yields are considerably low in India compared to the world average due to the unavailability of high yielding varieties and prevalence of biotic and abiotic stresses^5^. Cowpea being a self-pollinated crop, possess a very narrow genetic base which greatly restricted improvement endeavour, because the availability of genetic variations is an important prerequisite to initiate any breeding programme. Hence, a systematic breeding approach is required to circumvent the issue of low genetic variability and to generate improved cultivars associated with higher yielding capacity and stress resilience, which can significantly reduce the prevailing yield gap in cowpea.

Mutations are a necessary occurrence for evolution and hence for speciation and domestication of both plants and animals. Therefore, induced mutagenesis has been considered as a powerful tool to create contrast in traits of interest. Amongst the conventional and modern approaches of plant breeding, mutation breeding is a rapid, cost-effective and coherent method to accelerate the course of developing and screening crop genotypes with novel and improved agronomic traits^6^. With the advent of modern genomic techniques and advancement in molecular markers, the possibilities in mutation breeding are now amplified tremendously. In India, mutation breeding has led to the development and official release of only 9 mutant varieties of cowpea. This reflects the unexplored status of cowpea genetic resources and necessitates the use of mutation breeding for cowpea improvement^6^. In mutation breeding program, the assessment of mutagen induced genetic variability is imperative for the effective and efficient utilisation of genetic resources. For the accurate and quick assessment of genetic variability several morphological, physiological, biochemical and molecular markers are used. Although, the morphological and physiological markers based assessment of genetic variability is valuable for early field based characterization and selection but these markers are influenced by environmental flux to a great extent^7^. In order to validate the morphological and physiological marker based data, the study needs to be supplemented with more robust genetically linked molecular and biochemical markers for selection precision. Among the molecular markers SSRs and very recently developed CBDP markers are widely used to validate the morphological data^8^. The biochemical markers have been employed in several crops to assess the extent of genetic diversity^9^. Among the biochemical markers, SDS-PAGE shows better validity and ease for ascertaining genetic diversity of crop species and consequently it is employed in a wide range of crops for diversity analysis^10^. The SDS-PAGE profile of seed storage proteins has been used by several researchers for the assessment of genetic variability in cowpea^11^. However, the use of SDS-PAGE for the evaluation of genetic variability in the mutagenized populations of cowpea is very meagre. The introduction of molecular techniques equipped researchers to analyse genetic variability. Simple Sequence Repeats (SSRs) are unique among molecular markers having repeats of short (2–6 bp) DNA sequences and widely used in marker-assisted breeding and evaluation of diversity/polymorphism^12,13^. SSRs are highly polymorphic, co-dominant, reproducible, simple and inexpensive which make them better than other markers for the evaluation of genetic variation^14^. In the present study, we report the first time use of CBDP markers for the molecular characterisation of mutant varieties and evaluation of genetic diversity in the mutagenized population. The CBDP marker system is an outstanding approach for evaluation of genetic variability in the mutagenized population of cowpea. Earlier, CBDP markers have also been employed for the genetic diversity analysis, DNA fingerprinting, linkage mapping/QTLs and MAS in several crops such as cotton and jute^15^. CBDP markers are eighteen nucleotides long and harbour central CCAAT nucleotides with a consensus sequence GGCCAATCT located ~80 bp upstream of the start codon and filler sequence at the 5′ end and di- or trinucleotides at the 3′ end^16^.

Therefore, the objective of this study was to characterize the high yielding mutant genotypes generated through induced mutagenesis in two widely grown cowpea varieties of India, after continuous selections from M_2_ to M_4_ generations. The biochemical and molecular marker based analysis of divergence was done to assess the induced genetic deviations among the genotypes for comparative selection of mutants.

## Material and Methods

### Experimental methods

**Figure 1:**
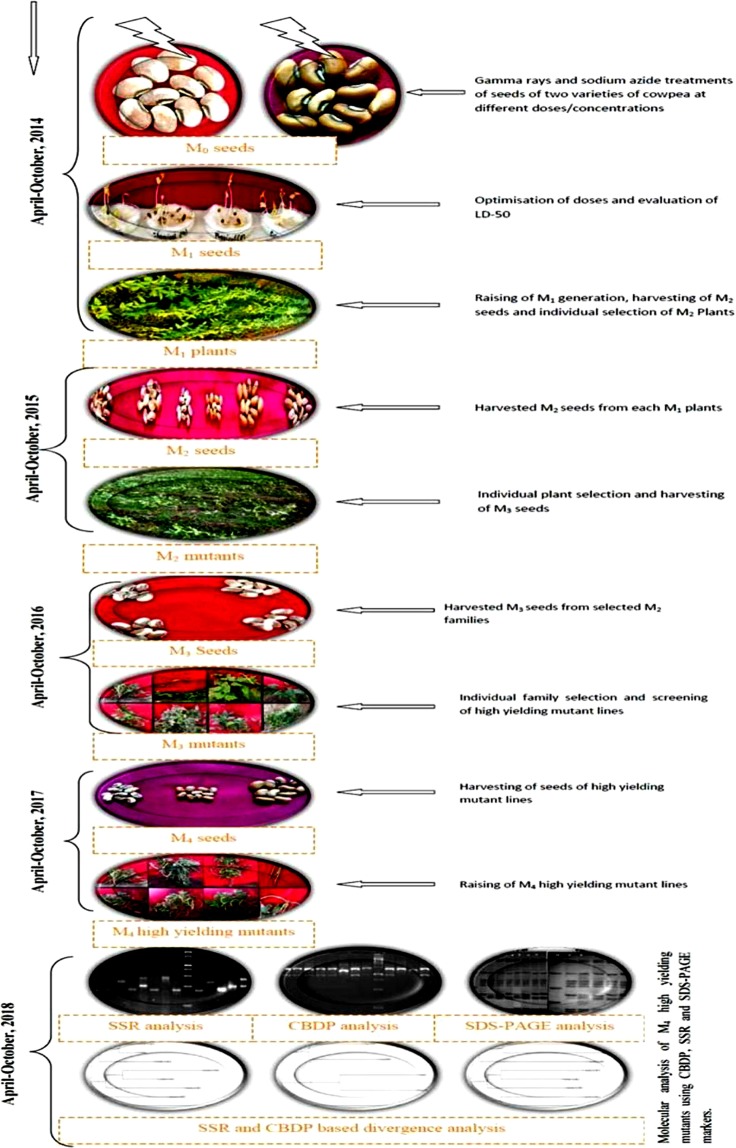
Illustration of the induced mutagenesis and selection methods during 2014–2018.

The experimental procedure followed in the present study is illustrated in Fig. 1 and have been adapted from Laskar and Khan^17^. The cowpea varieties Gomati VU-89 and Pusa-578 were procured from NBPGR, IARI, New Delhi, India. The dry and healthy seeds (moisture 10.0%) of both the varieties were directly irradiated with 100, 200, 300 and 400 Gy of gamma rays with Cobalt-60 radioisotope (^60^Co) at National Botanical Research Institute, Lucknow. For chemical treatments, seeds were pre-soaked in water for 6 hours and then treated with different doses (v/v) of sodium azide (SA) viz, 0.01%, 0.02%, 0.03% and 0.04% at room temperature of 25 ± 2 °C for 9 hrs. Also, a combination treatment sets of seeds viz. 100 Gy γ rays + 0.01% SA, 200 Gy γ rays + 0.02% SA, 300 Gy γ rays + 0.03% SA and 400 Gy γ rays + 0.04% SA, were prepared by directly treating the gamma rays treated seeds with sodium azide concentrations. Initially, the doses of chemical and physical treatments were optimised by calculating LD50 values based on germination rate and survival percentage. The 300 seeds from each treatment and control population were grown in 10 replications of 30 seeds each in the agricultural field of Aligarh Muslim University, Aligarh, India during mid-april 2014-October 2014 along with respective controls in a randomized complete block design (RCBD) to raise the M_1_ generation. Each block contained one replication from each of the 12 treatments and 1 control i.e., 13 plot (1.8 × 3 m) per block per variety. The seed to seed and row to row spacing was kept at 0.30 m and 0.60 m inside each block in the overall field size of 23.5 × 40 meter (Supp. Fig. 1A). All the seeds from M_1_ plants were harvested separately and 10 healthy M_2_ seeds from each harvested plant were sown treatment wise in plant progeny row basis for raising M_2_ generation during the mid-april 2015-October 2015. At each subsequent generation from M_2_ onwards, significantly stable and non-segregating mutant lines were advanced based on yield statistics. Based on the quantitative statistics for yield of M_2_ lines, 30 high yielding M_2_ mutant lines were isolated from each 100 Gy γ rays, 200 Gy γ rays, 0.01%SA, 0.02%SA, 100 Gy γ rays + 0.01% SA and 200 Gy γ rays + 0.02% SA treatment population, therefore, total 180 M_2_ mutant lines per variety were selected for selection in subsequent M_3_ generation. Subsequently, 10 M_3_ seeds from each of the selected 180 high yielding M_2_ mutant lines were considered for propagation into the M_3_ generation during the mid-april 2016-October 2016. In M_3_ generation seven and four high yielding mutant lines of Gomati VU-89 and Pusa-578, respectively were screened from 100 Gy γ rays, 200 Gy γ rays, 0.01% SA, 0.02%SA, 100 Gy γ rays + 0.01% SA, 200 Gy γ rays + 0.02% SA treatments based on highest seed yield potential and propagated to M_4_ generation during the mid-april 2017-October 2017 to check the stability of yield traits (Supp. Fig. 1B). To further narrow down the selections, only non-segregating mutant lines showing relatively stable yield trait were selected for characterizations. In M_4_ generation, seven high yielding mutant lines viz., Gomati VU-89-A (isolated at 0.02% SA), Gomati VU-89-B (isolated at 100 Gy γ rays + 0.01% SA), Gomati VU-89-C (isolated at 200 Gy γ rays), Gomati VU-89-D (isolated at 200 Gy γ rays + 0.02% SA), Gomati VU-89-E (isolated at 100 Gy γ rays), Gomati VU-89-F (isolated at 200 Gy + 0.02% SA) and Gomati VU-89-G (isolated at 100 Gy γ rays) were screened in var. Gomati VU-89 and four high yielding mutant lines viz., Pusa-578-A (isolated at 0.02% SA), Pusa-578-B (isolated at 200 Gy γ rays + 0.02% SA), Pusa-578-C (isolated at 200 Gy γ rays), Pusa-578-D (isolated at 100 Gy γ rays + 0.01% SA) screened in var. Pusa-578 (Supplementary Table 1) were subjected to statistical analysis for yield and yield attributing traits, physiological and biochemical traits to develop a relative profile of each mutant lines. Further, characterization using molecular markers was carried out to analyze the extent of genetic divergence induced among the mutant lines as well as the parent genotypes. The genotypic coefficient of variation (GCV), heritability (h^2^) and genetic advance (GA) of selected mutant lines have been evaluated to assess the stability and expressivity of the yield and yield attributing traits^18^. The genetic parameters were calculated for the traits to predict the extent of induced genetic variability. These parameters include the following:

$${\rm{GCV}}\,( \% )=\frac{\sqrt{{{\rm{\sigma }}}^{2}{\rm{g}}}}{\bar{{\rm{x}}}}\times 100$$

where, σ^2^ g (an estimate of genotypic variance) = (MSG − MSE)/r

MSG is an estimate of mean square of tested accession;

MSE is an estimate of mean square of error

r refers to the number of replications.

$${{\rm{h}}}^{2}( \% )=\frac{{{\rm{\sigma }}}^{2}{\rm{g}}}{{{\rm{\sigma }}}^{2}{\rm{p}}}\times 100$$

where σ^2^ g is the genotypic component of variance

σ^2^ p is the phenotypic component of variance.

$${\rm{GA}}={\rm{k}}.{\rm{\sigma }}{\rm{p}}.{{\rm{h}}}^{2}{\rm{bs}}$$

where, h^2^bs = Broad-sense heritability

σp refers to Phenotypic standard deviation of the mean performance of treated population

K = 2.64, constant for 1% selection intensity

### Nitrate reductase activity

The high yielding mutants were evaluated for Nitrate reductase activity (NRA) as per the protocol of Jaworski^19^. Leaves (200 mg) were chopped in 2.5 ml phosphate buffer (0.1 M, pH 7.4) (Appendix 1-A), 0.5 ml potassium nitrate (0.2 M) solution (Appendix 1-B), 2.5 ml isopropanol (5%) (Appendix 1-C) and were incubated for 2 hours at 30 °C in dark. 0.3 ml sulphanilamide solution (0.1%) (Appendix 1-D) and 0.3 ml NED-HCL (0.02%) (Appendix 1-E) were added to 0.4 ml incubated mixture. The mixture was diluted to 5 ml with double distilled water and the absorbance was read at 540 nm. The absorbance of each sample was compared with that of the calibration curve and NRA (nmol NO_2_.g^−1^.h^−1^FW) was computed on a fresh mass basis.

### Chlorophyll and carotenoid contents

The chlorophyll and carotenoid contents of leaves were estimated by the method of MacKinney^20^. About 1 g of finely cut fresh leaves was grounded to a fine pulp in 20 ml of acetone (Appendix 1-F). The mixture was centrifuged at 5,000 rpm for 5 minutes and the supernatant was collected in 100 ml volumetric flask. The residue was washed three times, using 80% acetone. The absorbance was read at 645 and 663 nm for chlorophyll and 480 and 510 nm for carotenoid against the acetone (80%) as blank. The chlorophyll and carotenoid contents present in the extracts of leaves was calculated according to the equation given by Arnon^21^.

$${\rm{Total}}\,{\rm{chlorophyll}}\,({\rm{mg}}.{{\rm{g}}}^{-1}\,{\rm{leaf}}\,{\rm{fresh}}\,{\rm{mass}})=\{20.2\,({{\rm{OD}}}_{645})+\,8.02\,({{\rm{OD}}}_{663})\}\times \frac{{\rm{V}}}{1000\times {\rm{W}}}$$

$${\rm{Carotenoid}}\,({\rm{mg}}.{{\rm{g}}}^{-1}\,{\rm{leaf}}\,{\rm{fresh}}\,{\rm{mass}})=\frac{7.6\,({{\rm{OD}}}_{480})-1.49\,({{\rm{OD}}}_{510})}{{\rm{d}}\times 1000\times {\rm{W}}}\times {\rm{V}}$$

where,

OD_645_, OD_663_, OD_480_, OD_510_ = Optical densities at respective wavelength

V = Volume of an extract

W = Mass of leaf tissues

d = Length of light path (d = 1.4 cm)

### Seed protein and mineral element contents

Seeds were pounded to the flour and 400 μl sample buffer was added to seed sample (0.01 g) as extraction buffer containing 0.5 M Tris-HCl (pH 6.8), 2.5% SDS, 10% glycerol and 5% 2-mercaptoethanol. Few drops of Bromophenol Blue was employed as a tracking dye to watch the movement of protein in the gel. Assessment of seed mineral elements such as iron (Fe), copper (Cu) and zinc (Zn) was carried out in an atomic absorption spectrophotometer^22^. The data were subjected to Duncan’s multiple range test to assess the significance degree.

### Sodium dodecyl sulphate polyacrylamide gel electrophoresis analysis

SDS-PAGE analysis was employed to characterize the protein pellets as per the protocol formulated by Laemmli^23^. Coomassie Brillant Blue G-250 die was added to 10% polyacrylamide gel and kept overnight. There are three main steps of SDS PAGE analysis:

#### Making SDS-PAGE gel

Ten per cent lower gel (separating gel) by adding the solutions (Appendix II-A) was prepared. A small layer of isopropanol to the top of the gel was added prior to polymerization for straightening the level of the gel. After the polymerisation of gel is complete, stacking gel (5%) was prepared by adding the solutions (Appendix II-B) and TEMED was added.

#### Sample preparation

The stored samples were thawed and vortex and diluted with ddH_2_O. Based on the Lowry Assay, the same amount of protein samples was prepared and mixed with the same volume of 2X protein sample buffer (Appendix II-C). The samples were boiled at 95 °C for 10 min. The samples were spanned at the maximal speed for 1 min in a tabletop centrifuge and left the samples at room temperature until ready to load onto the gel.

#### Electrophoresis

Electrophoresis running buffer (10×) (Appendix II-D) was poured into the opening of the casting frame between the gel cassettes. The region outside of the frame was filled with 10X running buffer. An equal amount (10 μl) of prepared protein samples was slowly loaded into each well and 10 μl of protein MW marker was also loaded. The standard protein molecular weight marker (Fermentas®), containing  five kinds of low molecular weight proteins was used. The established mold was then connected with the power supply and ran the gel at 50 mA until the dye reaches the bottom of the glass plate.

### Simple sequence repeat (SSR) marker analysis

The leaf DNA was extracted from 10-days-old seedlings using CTAB (Cetyl Trimethyl Ammonium Bromide) method as per Agbagwa *et al*.^24^ for SSR and CBDP analysis. 82 genic-SSR primers were screened and finally, nine primers lead to the generation of polymorphic bands (Sup. Table 2). Briefly, DNA amplification was carried out in a 96 well thermocycler in a volume of 20 μl each containing PCR grade water, 1.5–2 µl of 50 ng template DNA, 0.5ul of 10 µM of each primer, 0.5 µl of 10 mM of the dNTPs mix, 2 µl of 10X Taq buffer and 0.2 µl of Taq DNA Polymerase. The touchdown PCR was performed in a thermocycler following the program in which the first 5 cycles of 30 s at 94 °C, 45 s at 61 °C with a 1 °C decrease in annealing temperature per cycle and 1 min at 72 °C followed by 30 cycles at 30 s at 94 °C, 45 s at 57 °C and 1 min at 72 °C and a final extension at 72 °C for 10 min. The DNA bands were resolved on a 3% agarose gel stained with ethidium bromide, prepared by combination of MetaPhor and normal agarose in the ratio of 1:1 using 1X TBE buffer.

### CAAT box derived polymorphism marker analysis

The CBDP marker system is an excellent approach for assessment of genetic variability in the mutagenized population of cowpea. Previously, CBDP markers have been employed for the genetic diversity analysis, DNA fingerprinting, linkage mapping/QTLs and MAS in several crops^15^. However, these markers have never been used to assess the induced genetic diversity in any mutagenized population. 25 CBDP markers were screened and finally, nine primers lead to the generation of polymorphic bands (Sup. Table 3). We report first time applicability of CBDP markers in the mutant lines. The master mix consisted of 50 ng template DNA, 1X PCR buffer, 200 mM dNTPs, 0.5 μM CBDP primer and 1 U of Taq DNA polymerase in a total volume of 25.0 μl. The touchdown PCR was performed in a thermocycler following the program in which the first 5 cycles were run at 94 °C; 1 min for denaturing, 35 °C; 1 min for annealing, and 72 °C; 1 min for the extension. The annealing temperature was subsequently raised to 50 °C for another 35 cycles with a final extension for 10 min at 72 °C.

### Gel scoring and genetic divergent analysis

The SDS-PAGE protein, SSR and CBDP DNA bands were recorded as presence (1) or absence (0) and used to calculate Jaccard’s similarity coefficients and generate a dendrogram using PAST 3^25^. Per cent of polymorphism was determined as:

$$Percent\,of\,polymorphism=\frac{Number\,of\,polymorphic\,bands}{Total\,number\,of\,bands}\times 100$$

Polymorphism information content (PIC) value was determined as^26^.

$$PIC=1\,-\,\mathop{\sum }\limits_{n=1}^{f}{({P}_{ij})}^{2}$$

where, n is the number of marker alleles for marker i and Pij is the frequency of the jth allele for marker i.

## Results and Discussion

The M_4_ high yielding mutant lines revealed an ample range of variability for polygenic traits such as pods bearing branches, number of pods and seed yield per plant. The quantitative data and genetic data for different traits of mutant lines and their controls are provided in Tables 1 and 2. Statistically significant increase in the mean values of these quantitative traits was observed in the mutants over the control population of both varieties. Among the mutants of the var. Gomati VU-89, the maximum increase in seed yield (139.81 g) pods bearing branches (15.50) and pods per plant (78.20) was shown by the Gomati VU-89-G isolated at 100 Gy gamma rays treatment. In the var. Pusa-578, the highest seed yield (126.07 g), pods bearing branches (17.23) and pods per plant (55.00) were observed in the mutant Pusa-578-C isolated at 200 Gy gamma rays treatment. The results revealed increase in the GCV, h^2^ and GA in mutant lines Gomati VU-89-A, Gomati VU-89-B, Gomati VU-89-C, Gomati VU-89-D, Gomati VU-89-E, Gomati VU-89-F and Gomati VU-89-G and Pusa-578-A, Pusa-578-B, Pusa-578-C and Pusa-578-D compared to their respective control, which in turn suggests the selection for quantitative traits in the M_3_ generation was very successful. Heritability (h^2^) and genetic advance (GA) has been the most dependable statistical measure to ascertain the genetic stability of quantitative trait(s) in plant breeding. The significantly high heritability and genetic advance estimated in this study confirmed that the high yielding mutant lines were least influenced by the environment and were genotypically stable for further advancement.

**Table 1:** Estimates of range, mean values ($\bar{\mathrm{x}}$), shift in $\bar{\mathrm{x}}$ and genetic parameters for various quantitative traits of M_4_ high yielding mutants of cowpea var. Gomati VU-89.

Strain Number	Range	Mean^#^ ± S. E.	Shift in $\bar{x}$	GCV (%)	h^2^ (%)	GA (% of $\bar{{\rm{x}}}$)
**Pods bearing branches per plant**						
**Gomati VU-89**	7–11	8.86^f^ ± 0.14	0.00	7.23	45.12	12.82
Gomati VU-89-A	9–17	13.66^bc^ ± 0.35	+4.80	25.11	89.83	62.83
Gomati VU-89-B	9–17	12.96 ^cd^ ± 0.30	+4.10	24.26	92.64	61.65
Gomati VU-89-C	8–16	12.8^e^ ± 0.31	+3.94	23.88	90.12	59.86
Gomati VU-89-D	9–15	12.70^d^ ± 0.26	+3.84	19.29	88.11	47.80
Gomati VU-89-E	9–15	12.56^b^±0.28	+3.70	20.89	87.33	51.54
Gomati VU-89-F	10–18	13.93^b^ ± 0.29	+5.07	20.42	89.24	50.93
Gomati VU-89-G	10–20	15.50^a^ ± 0.38	+6.64	24.10	90.16	60.43
**Pods per plant**						
**Gomati VU-89**	55–64	60.86^e^ ± 0.42	0.00	3.66	54.74	7.16
Gomati VU-89-A	60–96	74.56^d^ ± 1.63	+13.70	21.85	91.47	55.18
Gomati VU-89-B	50–90	75.86^bc^ ± 1.60	+15.00	20.81	91.00	52.42
Gomati VU-89-C	59–89	76.83^a^ ± 1.49	+15.97	19.34	94.47	48.83
Gomati VU-89-D	60–90	77.33^b^ ± 1.52	+16.47	19.10	90.06	47.86
Gomati VU-89-E	60–88	76.43^bc^ ± 1.34	+15.57	17.55	91.72	44.38
Gomati VU-89-F	65–90	77.13 ^cd^ ± 1.20	+16.27	15.86	92.56	40.30
Gomati VU-89-G	61–90	78.20^ab^ ± 1.42	+17.34	18.86	93.46	48.16
**Seed yield per plant**						
**Gomati VU-89**	20.12–98.50	92.64 ^g^ ± 0.42	0.00	2.47	56.32	4.91
Gomati VU-89-A	100.23–140.85	120.04 ^f^ ± 1.48	+22.83	12.50	92.30	31.70
Gomati VU-89-B	102.45–133.65	123.92^ef^ ± 1.43	+31.28	13.10	97.55	34.17
Gomati VU-89-C	110.24–138.24	127.80^de^ ± 1.62	+35.16	14.08	95.75	36.38
Gomati VU-89-D	110.35–139.45	130.45 ^cd^ ± 1.42	+37.81	12.23	96.82	37.81
Gomati VU-89-E	108.65–141.45	133.21^bc^ ± 1.67	+40.58	14.08	96.95	36.61
Gomati VU-89-F	107.35–143.56	136.78^ab^ ± 1.77	+44.14	14.83	97.86	38.74
Gomati VU-89-G	110.35–148.45	139.81^a^ ± 1.82	+47.18	14.77	97.54	38.52

^#^Means within rows followed by the same letter is not different at the 5% level of significance based on the Duncan Multiple range test.

**Table 2:** Estimates of range, mean values ($\bar{{\rm{x}}}$), shift in $\bar{{\rm{x}}}$ and genetic parameters for various quantitative traits of M_4_ high yielding mutants of cowpea var. Pusa-578.

Strain number	Range	Mean^#^ ± S. E.	Shift in $\bar{{\rm{x}}}$	GCV (%)	h^2^ (%)	GA (% of $\bar{{\rm{x}}}$)
**Pods bearing branches per plant**						
**Pusa-578**	10–14	11.93^d^ ± 0.22	0.00	9.24	48.60	17.00
Pusa-578-A	12–20	16.20^b^ ± 0.38	+4.27	23.28	91.15	58.68
Pusa-578-B	11–20	15.36^bc^ ± 0.33	+3.43	19.77	86.40	48.52
Pusa-578-C	15–20	17.23^a^ ± 0.27	+5.30	15.41	89.44	38.49
Pusa-578-D	14–19	16.13^c^ ± 0.26	+4.20	13.56	82.39	32.51
**Pods per plant**						
**Pusa-578**	38–45	41.23^d^ ± 0.29	0.00	3.52	51.02	6.64
Pusa-578-A	40–58	50.06^b^ ± 0.91	+8.83	17.77	90.45	44.62
Pusa-578-B	42–64	53.03^a^ ± 0.81	+11.80	14.87	90.07	37.26
Pusa-578-C	43–63	55.00^a^ ± 0.87	+13.77	16.15	92.70	41.05
Pusa-578-D	38–55	47.10^c^ ± 0.71	+5.87	15.07	91.16	37.98
**Seed yield per plant**						
**Pusa-578**	72.45–84.78	80.29^c^ ± 0.53	0.00	3.01	45.05	5.34
Pusa-578-A	102.33–138.15	119.91^b^ ± 1.49	+39.62	12.60	92.14	31.94
Pusa-578-B	101.23–138.85	118.54^b^ ± 1.41	+38.25	12.26	92.88	31.20
Pusa-578-C	109.25–137.24	126.07^a^ ± 1.49	+45.78	12.75	95.05	32.82
Pusa-578-D	102.45–130.45	122.08^b^ ± 1.20	+41.80	11.24	97.73	29.33

^#^Means within rows followed by the same letter is not different at the 5% level of significance based on the Duncan Multiple Range Test.

### NRA, chlorophyll and carotenoid contents

Compared to controls substantial increase in NRA, chlorophyll and carotenoid contents was recorded in the mutant lines Table 3. The maximum NRA (684.59 and 680.44 in nmol h^−1^ g^−1^ FW) was recorded for the mutants Gomati VU-89-G and Pusa-578-C, respectively. The correlation studies reflected a positive relationship of NRA with the yield and similar correlations have been reported in lentil^27^. Likewise, a statistically significant increase in the chlorophyll and carotenoid contents were also observed in the M_4_ mutants of cowpea. Average chlorophyll contents revealed a significant rise to 2.47 mg g^−1^ FW (Gomati VU-89-G) and 2.68 mg g^−1^ FW (Pusa-578-C) against the controls of Gomati VU-89 and Pusa-578 with mean values of chlorophyll contents as 2.05 and 2.55 mg g^−1^ FW, respectively. The close correlation between pigment contents and seed yield as recorded in M_4_ high yielding mutants has also been reported in wheat^28^. Carotenoids are precursors of vital vitamins and are essential antioxidants that reduce the oxidative stress to a greater extent^29^. Similarly, carotenoid contents also reflected a significant increase up to 0.84 and 0.91 mg g^−1^ FW in the Gomati VU-89-G and Pusa-578-C, respectively. Enhancement in carotenoid contents in the mutants as recorded in the present study in cowpea play a vital role in promoting the plant health and also possesses protective health benefits for humans and livestock; hence, development of high carotenoid varieties is an emerging area of interest for mutation breeders. The high carotenoid content mutants were isolated from gamma irradiated seeds, as the gamma rays induced oxidative stress in the irradiated material it can be concluded that carotenoid synthesizing genes might be overexpressed in these mutant lines due to the plant’s natural defence mechanisms. The increased carotenoid contents in response to gamma rays induced oxidative stress ultimately enhanced the normal plant growth and subsequent fixation of the micromutations. Hence, considerable augmentation in the carotenoid contents in M_4_ high yielding mutants may be attributed to the mutagen caused hypoactivity or hyperactivity of carotenoid biosynthesis genes. The mutagen induced up-regulation of carotenoid biosynthesis genes may impart tolerance to a wide range of climate induced stresses such as drought and salinity^28^. EMS induced *Arabidopsis thialiana* and *Hordeum vulgare* mutants tolerant to the paraquat and imidazolinone herbicide respectively were successfully isolated by Gechev *et al*.^30^ and Lee *et al*.^31^ using induced mutagenesis. Hence, the studies revealed that the high yielding mutants may also be tolerant to a range of environmental flux and can be identified, screened and allowed for after trait fixation in advanced generations.

**Table 3:** Estimates of mean values ($\bar{{\rm{x}}}$), shift in $\bar{{\rm{x}}}$ and coefficient for variation for nitrate reductase activity (nmol h^−1^ g^−1^ FW), chlorophyll (mg g^−1^ FW) and carotenoid (mg g^−1^ FW) contents in the leaves of M_4_ high yielding mutants of two varieties of cowpea.

Strain number	NRA			Chlorophyll			Carotenoid		
	Mean^#^ ± S. E.	Shift in $\bar{{\rm{x}}}$	C.V. (%)	Mean^#^ ± S. E.	Shift in $\bar{{\rm{x}}}$	C.V. (%)	Mean^#^ ± S. E.	Shift in $\bar{{\rm{x}}}$	C.V. (%)
**Gomati VU-89**	630.82^e^ ± 1.28	0.00	0.35	2.05^e^ ± 0.01	0.00	1.23	0.59^d^ ± 0.01	0.00	1.69
Gomati VU-89-A	655.11^d^ ± 5.01	24.29	1.32	2.30^bc^ ± 0.03	0.15	2.19	0.69^c^ ± 0.03	0.10	6.50
Gomati VU-89-B	660.64 ^cd^ ± 2.32	29.82	0.61	2.21^d^ ± 0.03	0.06	2.04	0.73^bc^ ± 0.01	0.14	2.86
Gomati VU-89-C	669.40^bc^ ± 4.68	38.58	1.21	2.36^b^ ± 0.02	0.21	1.27	0.74^bc^ ± 0.02	0.15	4.87
Gomati VU-89-D	666.82^bc^ ± 1.80	36.00	0.47	2.27^c^ ± 0.01	0.12	1.11	0.75^bc^ ± 0.03	0.16	7.31
Gomati VU-89-E	662.69 ^cd^ ± 4.40	31.87	1.15	2.30^bc^ ± 0.01	0.15	0.90	0.78^ab^ ± 0.01	0.19	2.66
Gomati VU-89-F	674.13^b^ ± 2.90	43.31	0.74	2.16^d^ ± 0.01	0.01	0.93	0.77^b^ ± 0.01	0.18	2.69
Gomati VU-89-G	684.59^a^ ± 3.30	53.77	0.84	2.47^a^ ± 0.02	0.32	1.46	0.84^a^ ± 0.02	0.25	3.65
**Pusa-578**	652.15^c^ ± 1.95	0.00	0.52	2.55^c^ ± 0.01	0.00	0.99	0.68^c^ ± 0.01	0.00	2.24
Pusa-578-A	668.02^b^ ± 4.43	37.20	1.15	2.64^a^ ± 0.02	0.49	1.16	0.84^a^ ± 0.02	0.15	3.62
Pusa-578-B	665.97^b^ ± 4.77	35.15	1.24	2.58^b^ ± 0.02	0.43	1.16	0.89^a^ ± 0.01	0.20	1.71
Pusa-578-C	680.44^a^ ± 2.71	49.62	0.69	2.68^ab^ ± 0.01	0.53	0.94	0.91^b^ ± 0.01	0.22	2.78
Pusa-578-D	667.15^ab^ ± 3.58	36.33	0.93	2.65^ab^ ± 0.01	0.50	0.58	0.80^b^ ± 0.02	0.11	3.80

FW = Fresh Weight; ^#^Mean within columns followed by the same letter is not different at the 5% level of significance based on Duncan Multiple Range Test.

### Seed protein and mineral element contents

Induced mutagenesis for the fortification of nutritional values, particularly proteins and micronutrients, has been an emerging field of the crop improvement programme. The M_4_ high yielding mutants evaluated for micronutrient and protein quantity to ascertain the opportunity of an increase in yield coupled fortification of micronutrients. Selections were exclusively based on the quantitative traits up to the M_4_ generation, any augmentation in the nutrient contents in M_4_ high yielding mutants is an added value to the mutagenesis research. The average values and coefficient of variation (C.V.%) for the proteins and minerals of M_4_ mutants have been estimated (Table 4). In the var. Gomati VU-89 a slight but significant improvement in seed protein contents for the mutants Gomati VU-89 A, B and E and insignificant improvement for mutants Gomati VU-89-C, D, F and G, and substantial improvement for all the mutants isolated from the var. Pusa-578 was recorded. The maximum increase in protein quantity was recorded in the Gomati VU-89-E (23.16%) in comparison to the control of the var. Gomati VU-89 (22.28%). In the var. Pusa-578, the maximum increase (23.00%) in the average protein contents was recorded in the Pusa-578-D compared to 21.27% in the control of the var. Pusa-578. Similar reports on high yielding mutants with high protein content have been reported in various crops by Tomlekova^32^ and Laskar *et al*.^27^ while Rehman *et al*.^33^ reported the contrary results of high yield with high protein content. Lal and Tomer^34^ is of the view that the augmented NRA might be the reason for the improved protein contents of mutants of M_4_ high yielding mutants. Increased NRA in the mutants may also be attributed to the increased protein content in seeds in the mutants isolated in present experimentation. Genetic and environment interactions also influence the total protein quantity in seeds^35^.

**Table 4:** Mean and coefficient of variation for total seed protein (%), iron, zinc and copper contents (mg 100 g^−1^) of M_4_ high yielding mutants of cowpea.

Strain number	Protein		Iron		Copper		Zinc	
	Mean^#^ ± S. E.	C.V. (%)	Mean^#^ ± S. E.	C.V. (%)	Mean^#^ ± S. E.	C.V. (%)	Mean^#^ ± S. E.	C.V. (%)
**Gomati VU-89**	22.28^b^ ± 0.17	1.31	8.32^d^ ± 0.08	0.92	1.85^e^ ± 0.02	1.90	2.84^d^ ± 0.02	1.07
Gomati VU-89-A	23.10^a^ ± 0.02	0.17	8.97^b^ ± 0.09	1.70	2.30^bc^ ± 0.03	2.19	3.32^c^ ± 0.06	3.26
Gomati VU-89-B	23.15^a^ ± 0.06	0.43	9.32^a^ ± 0.06	1.21	2.21^d^ ± 0.03	2.04	3.17^c^ ± 0.02	1.31
Gomati VU-89-C	22.65^b^ ± 0.27	0.93	8.74^bc^ ± 0.06	1.26	2.16^d^ ± 0.01	0.93	3.36^ab^ ± 0.03	1.49
Gomati VU-89-D	22.67^b^ ± 0.02	0.16	8.38^d^ ± 0.09	1.78	2.27^c^ ± 0.01	1.11	3.46^a^ ± 0.03	1.59
Gomati VU-89-E	23.16^a^ ± 0.03	0.24	9.24^a^ ± 0.07	1.25	2.30^bc^ ± 0.01	0.90	3.10^c^ ± 0.04	2.33
Gomati VU-89-F	22.46^b^ ± 0.06	0.47	8.48^d^ ± 0.12	2.45	2.37^a^ ± 0.02	1.52	3.17^c^ ± 0.03	1.38
Gomati VU-89-G	22.46^b^ ± 0.06	0.50	8.52 ^cd^ ± 0.06	1.23	2.36^ab^ ± 0.02	1.27	3.19^c^ ± 0.03	1.72
**Pusa-578**	21.27^b^ ± 0.13	1.06	8.17^d^ ± 0.04	0.83	1.75^d^ ± 0.01	0.87	2.74^d^ ± 0.02	0.97
Pusa-578-A	22.43^a^ ± 0.33	2.55	8.53^bc^ ± 0.06	1.29	2.24^a^ ± 0.01	1.03	3.07^c^ ± 0.03	1.64
Pusa-578-B	22.48^a^ ± 0.38	2.96	8.75^ab^ ± 0.08	1.49	2.18^ab^ ± 0.02	1.40	3.15^bc^ ± 0.03	0.97
Pusa-578-C	22.85^a^ ± 0.16	1.21	8.48^c^ ± 0.09	1.91	2.11^c^ ± 0.03	2.07	3.20^b^ ± 0.03	1.57
Pusa-578-D	23.00^a^ ± 0.31	2.36	8.78^a^ ± 0.07	1.45	2.17^b^ ± 0.02	1.38	3.33^a^ ± 0.06	2.92

^#^Mean within columns followed by the same letter is not different at the 5% level of significance based on Duncan Multiple Range Test.

The estimation of micronutrients resulted in a slight but significant increase in the mean seed protein contents in Gomati VU-89-A, B and E and Pusa-578-A, B, C and D mutant lines. The enhancement in the seed micronutrient contents of pulses through mutation breeding could purge the micronutrient deficiencies across the globe^36^. A positive deviation from the control means was recorded in the seeds of mutants for the estimated average values for the mineral elements such as Fe, Zn and Cu. The largely significant enhancement in the iron contents (9.32 and 8.78 mg 100 g^−1^) was estimated in the mutants Gomati VU-89-B and Pusa-578-D, respectively. The mutants Gomati VU-89-D (3.46 mg 100 g^−1^) and Pusa-578-D (3.33 mg 100 g^−1^) while as Gomati VU-89-F (2.37 mg 100 g^− 1^) and Pusa-578-A (2.24 mg 100 g^−1^) were found to contain maximum zinc and copper contents, respectively. The results were in agreement with the findings in soybean mutants induced by gamma rays^37^. Tomlekova *et al*.^38^ also reported the increased concentration of minerals such as iron, copper and zinc in pepper mutants with higher β-carotene contents. Hence, it can be concluded that the increase in the mineral elements might be due to the augmentation of β-carotene contents in the M_4_ high yielding mutants. Even though there are several reports of mutagen induced variations in quantitative traits of cowpea but the literature is very scanty on the mutagen induced variation in Fe, Zn and Cu contents. The results confirmed the scope of isolating mutant lines with increased micronutrients content coupled with a higher yield.

### SDS-PAGE analysis

**Figure 2:**
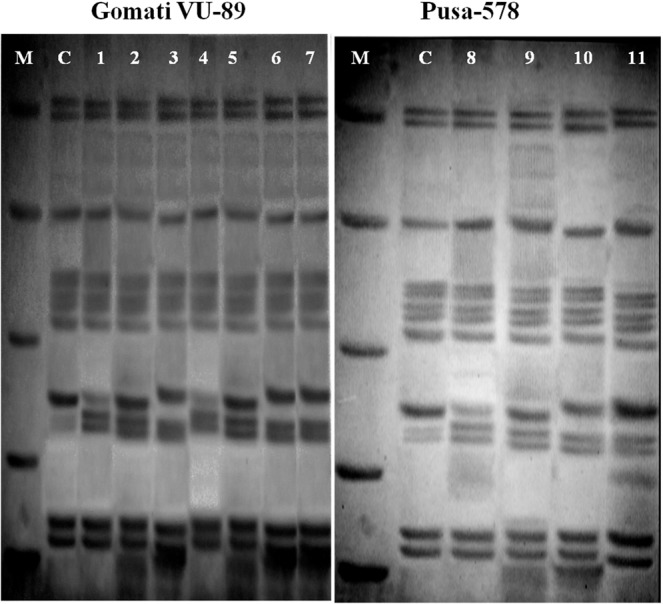
SDS PAGE profile showing bands of proteins of control and M_4_ high yielding mutants of cowpea varieties Gomati VU-89 and Pusa-578. Lane1: Marker (M); Lane2: Control (Gomati VU-89) (C); Lane3: Gomati VU-89-A (1); Lane4: Gomati VU-89-B (2); Lane5: Gomati VU-89-C (3); Lane6: Gomati VU-89-D (4); Lane7: Gomati VU-89-E (5); Lane8: Gomati VU-89-F (6); Lane9: Gomati VU-89-G (7); Lane1: Marker (M); Lane2: Control (Pusa-578) (C); Lane3: Pusa-578-A (8); Lane4: Pusa-578-B (9); Lane5: Pusa-578-C (10); Lane6: Pusa-578-D (11).

**Figure 3:**
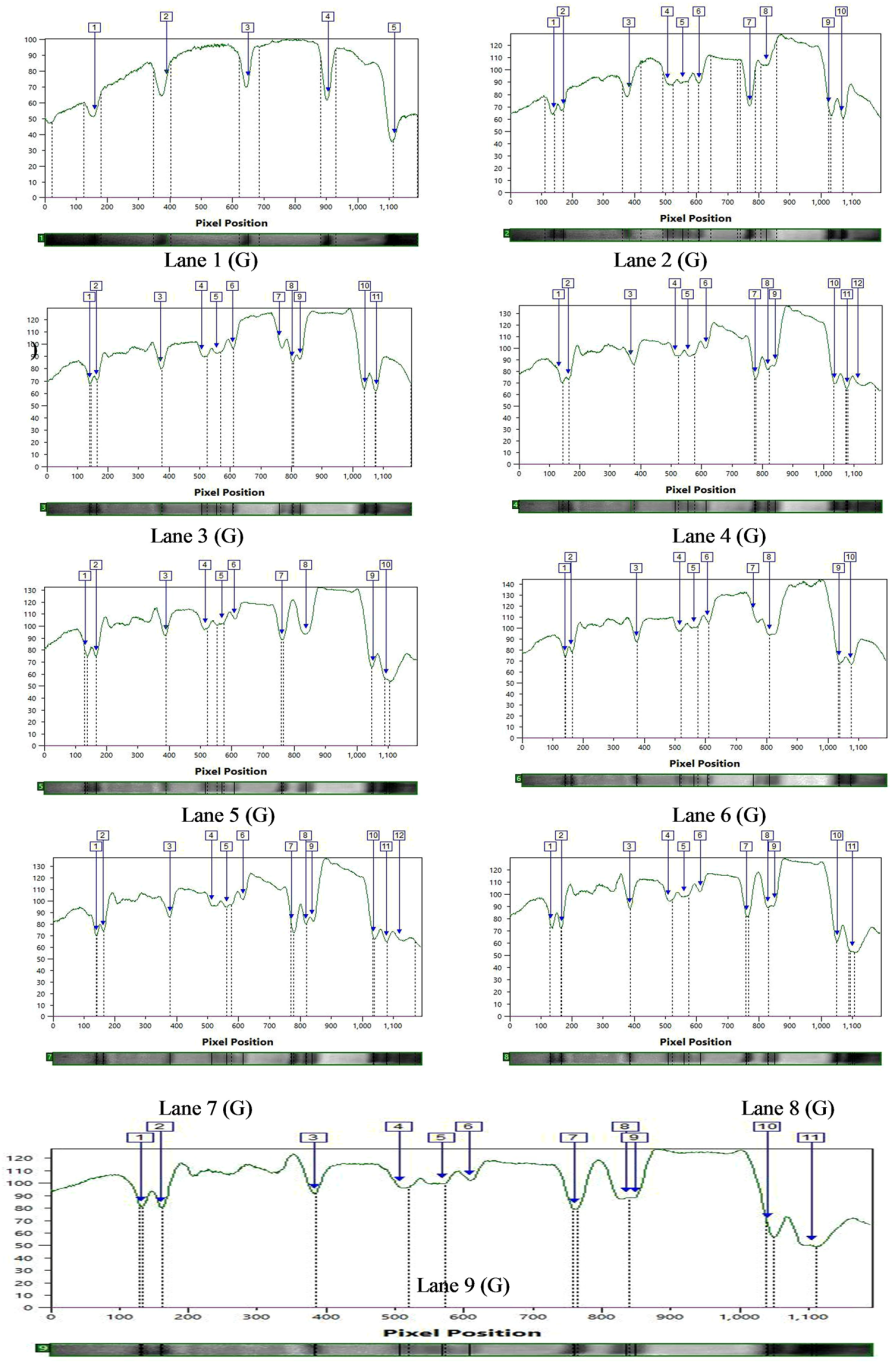
SDS PAGE band intensity curves showing different resolved proteins of controls and M_4_ high yielding mutants of cowpea var. Gomati VU-89. Lane1(G)- Marker (M); Lane 2 (G)- Control (Gomati VU-89); Lane 3(G)- Gomati VU-89-A; Lane 4(G)- Gomati VU-89-B; Lane 5(G)- Gomati VU-89-C; Lane 6(G)- Gomati VU-89-D; Lane7(G)- Gomati VU-89-E; Lane 8(G)- Gomati VU-89-F; Lane 9(G)- Gomati VU-89-G.

**Figure 4:**
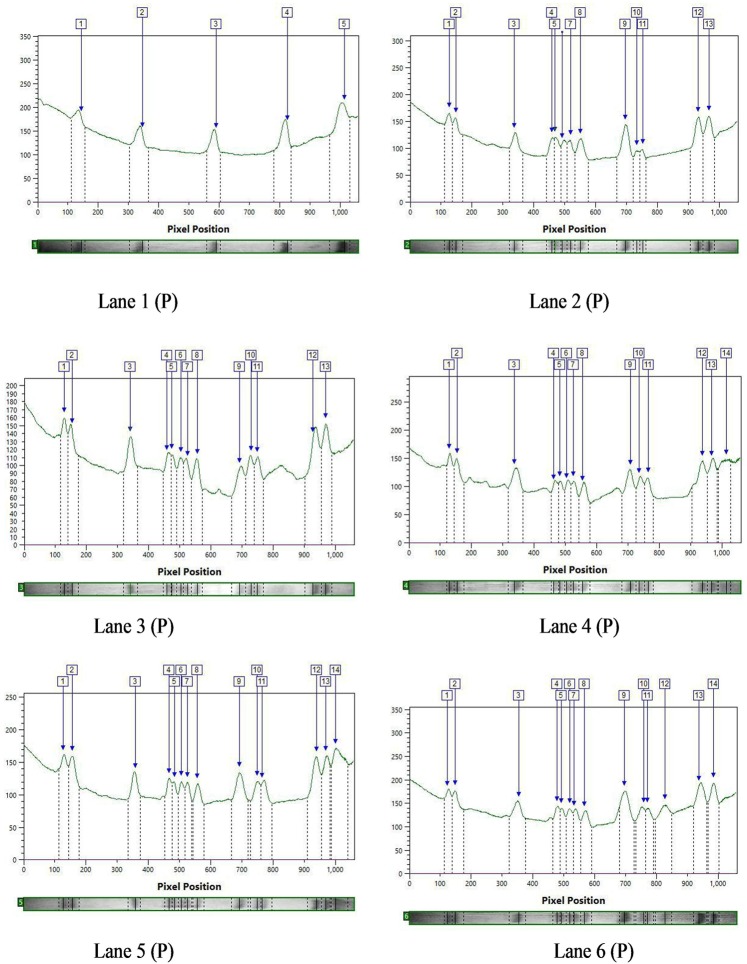
SDS PAGE band intensity curves showing different resolved proteins of controls and M_4_ high yielding mutants of cowpea var. Pusa-578. Lane 1(P)- Marker (M); Lane 2 (P)- Control (Pusa-578); Lane 3 (P)- Pusa-578-A; Lane 4 (P)- Pusa-578-B; Lane 5 (P)- Pusa-578-C; Lane 6 (P)- Pusa-578-D.

**Figure 5:**
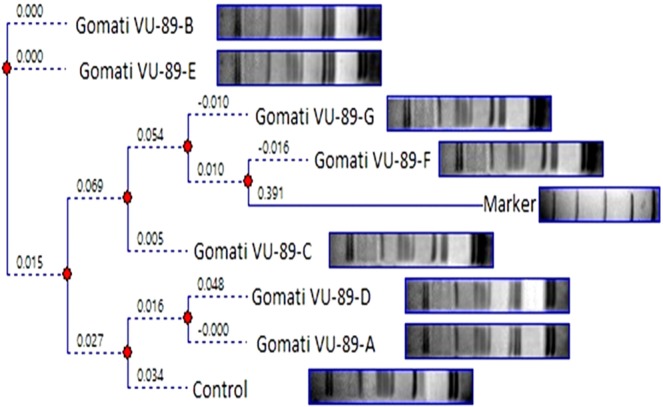
Dendrogram analysis of control and seven M_4_ high yielding mutants based on SDS PAGE profile of protein sample of cowpea var. Gomati VU-89.

**Figure 6:**
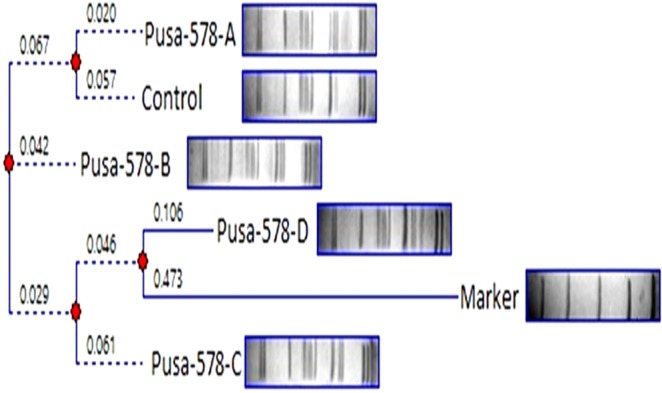
Dendrogram analysis of control and four M_4_ high yielding mutants based on SDS PAGE profile of protein sample of cowpea var. Pusa-578.

The SDS-PAGE profile of seed proteins revealed variations in the number and intensity of protein bands of mutant lines (Figs. 2–4). These polymorphic changes may be attributed to either mutagen induced variations in the DNA sequences that code for the proteins or genes involved in post-transcriptional modifications. The calculated molecular weight (kDa) of the protein bands of controls and M_4_ cowpea mutants are given in Tables 5 and 6. In the var. Gomati VU-89, of the total 87 bands of different MW generated, 54 bands were found to be polymorphic and 33 bands were monomorphic, which led to total polymorphism percentage of 62.06%. In the var. Pusa-578, 28 bands were polymorphic and 40 bands were monomorphic, of the total 68 bands generated, which resulted in total polymorphism percentage of 41.17% (Table 7). Mutants selected from var. Gomati VU-89 showed more expression and polymorphism between 45.45–103.53 kDa proteins. The polymorphism and more expression can also be confirmed by lane and band analysis and dendrogram (Figs. 3–6). Laskar *et al*.^27^ reported substantial genetic divergence between and within mutants of *Lens culinaris* which were not always reflected at the phenotypic level. Laskar *et al*.^27^ further reported that SDS-PAGE analysis of seed protein could be employed as an efficient means for screening mutants that cannot be identified solely by morphological data alone. Nonetheless, in conformity with the results reported by Laskar *et al*.^27^ on *Lens*, we were able to make use of SDS-PAGE as a tool for determining genetic relationships within a mutagenized population of *Vigna unguiculata*. The variations in the electrophoresis patterns of the seed proteins after mutagen treatments have also been reported in various pulse crops^35–39^. Hong *et al*.^40^ advocated that the response of mutants towards a wide range of environmental stresses can also be determined using SDS-PAGE analysis and would be useful in screening the stress tolerant mutants with high yield. Belele *et al*.^41^ in *Phaseolus vulgaris*, Auti and Apparao^42^ in *Vigna radiata*, Nakagawa *et al*.^43^ in *Glycine max*, Khadke and Kothekar^44^ in *Vigna acconitifolia*, Kozgar *et al*.^45^ in *Cicer arietinum*, Khursheed and Khan^46^ in *Vicia faba* and Laskar and Khan^17^ in *Lens culinaris* also noticed mutagen induced variations in the electrophoresis patterns of the seed storage proteins. The unique pattern of protein bands may also serve as markers for screening mutant lines. In general, the appearance of mutagen induced new bands with varied intensities reveals the new protein expression that might be attributed to the higher yield in mutant lines.

**Table 5:** Molecular weight (kDa) of different protein bands in SDS-PAGE profile of control and M_4_ high yielding mutants of cowpea var. Gomati VU-89.

Bands	Lane 1: Marker	Lane 2: Gomati VU-89 (Control)	Lane 3: Gomati VU-89-A	Lane 4: Gomati VU-89-B	Lane 5: Gomati VU-89-C	Lane 6: Gomati VU-89-D	Lane 7: Gomati VU-89-E	Lane 8: Gomati VU-89-F	Lane 9: Gomati VU-89-G
	MW	MW	MW	MW	MW	MW	MW	MW	MW
01	97.4	100.15	100.15	101.53	101.53	100.15	100.15	101.53	101.53
02	66.0	96.06	97.10	97.10	96.65	97.67	97.10	96.65	97.67
03	43.0	66.70	68.14	68.51	66.00	67.66	67.30	66.35	66.82
04	29.0	53.97	54.06	53.45	53.18	53.18	53.45	53.80	54.06
05	20.1	49.94	49.94	49.94	48.85	49.39	49.39	49.63	48.85
06		45.95	45.95	45.45	45.95	46.16	45.45	45.66	45.95
07		35.78	36.39	35.56	36.39	36.55	35.78	36.39	36.39
08		32.99	34.07	33.35	32.29	33.71	33.35	32.79	32.44
09		23.83	32.79	32.09	22.79	23.41	32.29	31.60	31.74
10		22.09	23.24	23.41	21.07	21.81	23.41	22.79	23.24
11			21.64	21.64			21.64	20.79	20.62
12				20.22			19.93

**Table 6:** Molecular weight (kDa) of different protein bands in SDS-PAGE profile of control and M_4_ high yielding mutants of cowpea var. Pusa-578.

Bands	Lane 1: Marker	Lane 2: Pusa-578 (Control)	Lane 3: Pusa-578-A	Lane 4: Pusa-578-B	Lane 5: Pusa-578-C	Lane 6: Pusa-578-D
	MW	MW	MW	MW	MW	MW
01	97.4	100.19	99.73	99.73	100.19	100.66
02	66.0	96.72	95.71	95.71	95.21	96.72
03	43.0	67.18	66.78	67.18	64.34	64.84
04	29.0	53.47	53.47	53.19	52.82	51.73
05	20.1	52.55	52.00	51.46	51.20	50.59
06		50.59	49.57	49.57	49.32	48.25
07		48.25	47.77	47.77	47.77	47.22
08		45.83	45.31	45.53	45.31	44.64
09		36.13	36.31	35.36	36.31	36.13
10		33.91	34.08	33.74	33.02	32.52
11		32.85	33.02	32.19	32.19	31.81
12		23.89	24.03	23.61	23.47	28.89
13		22.28	22.14	22.28	22.14	23.61
14				20.14	20.73	21.61

**Table 7:** Analysis of SDS banding pattern based on MW in M_4_ high yielding mutants of cowpea varieties Gomati VU-89 and Pusa-578.

Variety	TB	TPB	TMB	Polymorphism %
Gomati VU-89	87	54	33	62.06
Pusa-578	68	28	40	41.17

TB-Total bands, TPB-Total Polymorphic bands, TMB-Total monomorphic bands.

### Simple sequence repeat marker analysis

Even though protein markers play a significant role in the assessment of genetic diversity in the mutagen treated plants, however, DNA markers are more effective and have wide acceptance for studying the genetic divergence induced by mutagens. In addition, the interaction of mutagen with the DNA leads to the altered expression of genes and variations in protein profiles, hence, DNA markers divulge more precise state of gene mutations. As per the suggestions of Kumar *et al*.^47^ most of the agro-economical traits, particularly yield and yield attributes are governed by small additive effects of multiple genes and environmental factors, hence, to screen and isolate mutants with better traits, there is a need to integrate marker assisted selection (MAS). MAS has huge potential to enhance the efficiency and accuracy of traditional breeding approaches^48^. MAS has been widely employed for the assessment of genetic diversity due to the availability of a huge number of DNA markers^49^. Among the DNA markers, simple sequence repeats (SSRs) have vast distribution in both non-coding and coding sequence as tandem repeats of 1–6 DNA nucleotide motifs^50^.

**Figure 7:**
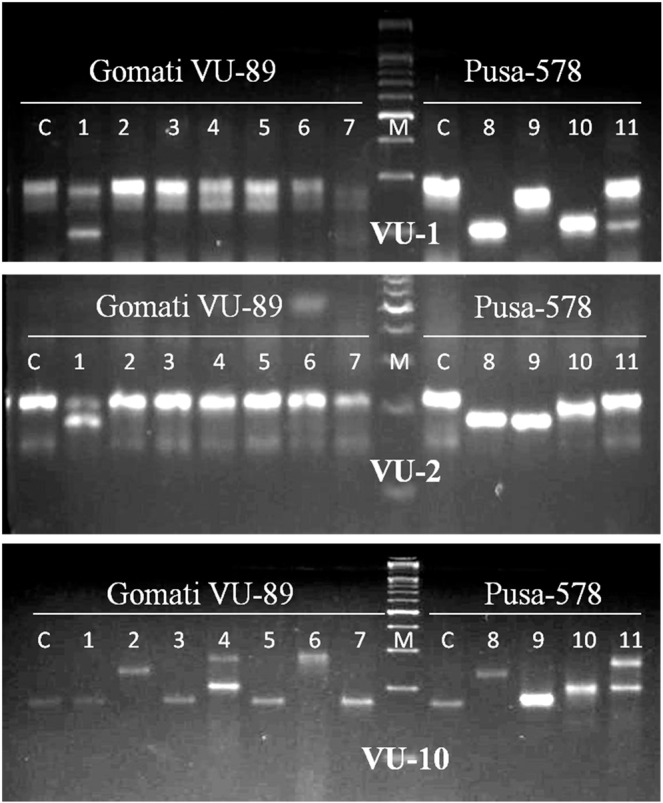
Cropped Gel profiles of markers generated using Simple sequence repeat primers. Picture a, b and c are showing markers generated using SSR primers VU-1, VU-2 and VU-10 respectively in control and M_4_ high yielding mutants of cowpea varieties Gomati VU-89 and Pusa-578. The full-length gels are presented in Supplementary Figs. II and III.

**Figure 8:**
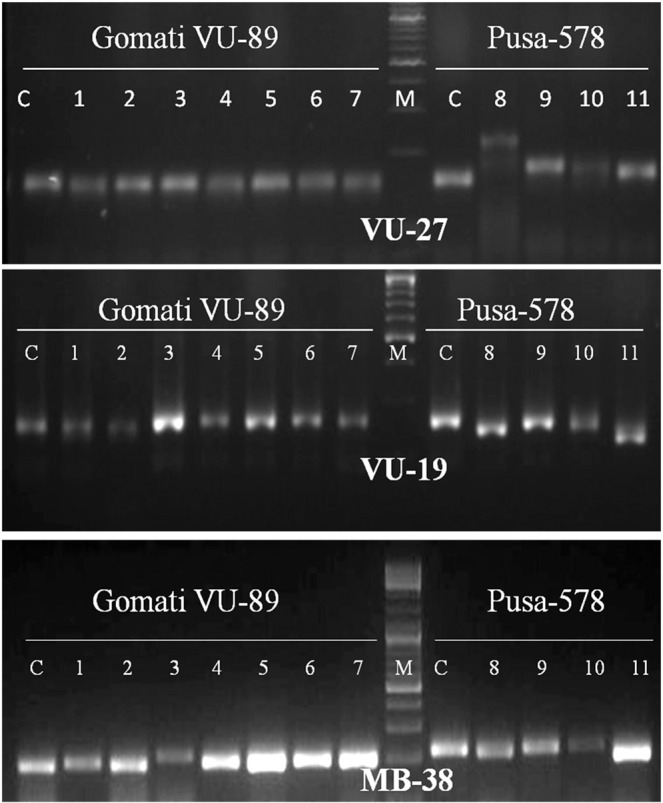
Cropped Gel profiles of markers generated using Simple sequence repeat primers. Picture a, b and c are showing markers generated using SSR primers VU-27, VU-19 and MB-38 respectively in control and M_4_ high yielding mutants of cowpea varieties Gomati VU-89 and Pusa-578. The full-length gels are presented in Supplementary Figs. II and V.

**Figure 9:**
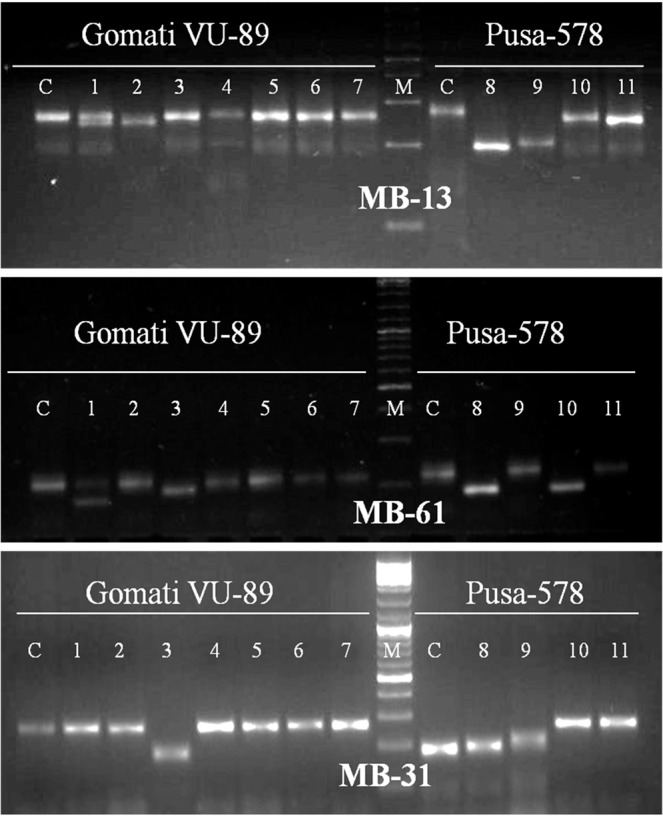
Cropped Gel profiles of markers generated using Simple sequence repeat primers. Picture a, b and c are showing markers generated using SSR primers MB-13, MB-61 and MB-31 respectively in control and M_4_ high yielding mutants of cowpea varieties Gomati VU-89 and Pusa-578. The full-length gels are presented in Supplementary Figs. IV, V and VI.

Genic-SSR primers developed earlier were employed for molecular characterization and assessment of genetic diversity of M_4_ high yielding mutants of cowpea. Earlier SSR markers have been employed for the assessment of genetic diversity of cultivated and wild cowpea species^51^, however, the literature is scanty on the assessment of genetic diversity of the mutagenized population using SSR markers. Out of 82 genic-SSR primers used, 9 primers generated polymorphic bands, 53 primers generated monomorphic bands and the remaining 20 primers failed to amplify DNA fragments. The selected 9 primers amplified 76 bands with 16 bands being polymorphic in the var. Gomati VU-89 and 47 bands with 24 bands being polymorphic in the var. Pusa-578 (Table 8; Figs. 7–9; Supp. Figs. II–VI). The apparent polymorphic bands in mutants revealed the broadening of the genetic base of the parental genotypes Gomati VU-89 and Pusa-578 induced by the mutagens. The maximum number of polymorphic amplified bands were 3 (VU-1, VU-10, MB-38) with an average of 1.78 in the var. Gomati VU-89 and 4 (VU-10, VU-27) with an average of 2.67 in the var. Pusa-578. The values of polymorphism were between 37.50% (MB-38) and 11.00% (VU-27; VU-19) with an average of 20.19 in the var. Gomati VU-89 and 80.00% (VU-27) and 20.00% (MB-38) with an average of 50.74% in the var. Pusa-578. The maximum PIC values were 0.83 (VU-10) with an average of 0.64 in the var. Gomati VU-89 and 0.83 (VU-10) with an average of 0.72 in the var. Pusa-578, respectively. Most of the genic-SSR markers with a PIC value of more than 0.5 are considered very informative for studying polymorphism study in the selected mutants. Among the primers, VU-27 primer resulted in maximum polymorphism (80.00%), hence, the control and mutant population can be differentiated using the VU-27 primer. In general, SSRs are PCR based DNA markers with better precision as it is evident from any change in the number and/or size of microsatellite repeats at a locus among individuals led to the formation of different sized bands^52^. SSR markers are very informative, multi-allelic, co-dominant, reproducibility, abundance and wide genomic distribution and hence are preferred over other molecular markers for assessment of genetic diversity. SSRs are also amenable to large-scale genotyping and hence suitable for construction of high-density genome maps, gene/QTL mapping and marker-assisted selection^53^.

**Table 8:** Details of genic-SSR primer, GC content, total bands generated and polymorphic information for each primer.

		Gomati VU-89				Pusa-578			
Primer ID	GC %	TB	TPB	Polymorphism %	PIC	TB	TPB	Polymorphism %	PIC
**VU-1**	50.0	9	3	33.33	0.69	6	3	50.00	0.80
**VU-2**	47.65	9	1	11.11	0.37	5	3	60.00	0.76
**VU-10**	49.52	9	3	33.33	0.83	6	4	66.66	0.83
**VU-27**	44.26	8	1	11.00	0.59	5	4	80.00	0.76
**VU-19**	50.0	8	1	11.00	0.59	5	2	40.00	0.76
**MB-31**	48.27	8	1	11.11	0.58	5	3	60.00	0.76
**MB-13**	49.65	8	1	11.11	0.59	5	2	40.00	0.59
**MB-61**	43.65	9	2	22.22	0.76	5	2	40.00	0.59
**MB-38**	46.52	8	3	37.5	0.80	5	1	20.00	0.59
**Total**	—	76	16	181.71	5.80	47	24	456.66	6.44
**Average**	—	8.44	1.78	20.19	0.64	5.22	2.67	50.74	0.72

TB-Total number of bands, TPB-Total polymorphic bands, PIC-Polymorphic information content.

### CAAT box derived polymorphism marker analysis

**Figure 10:**
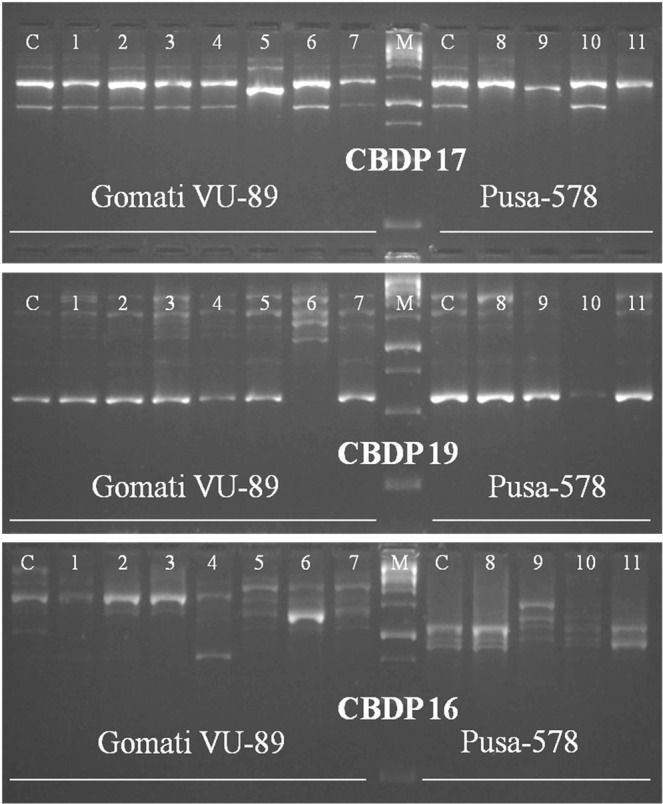
Cropped Gel profiles of markers generated using CAAT box derived primers. Picture a, b and c are showing markers generated using CBDP primers CBDP 17, 19 and 16 respectively in control and M_4_ high yielding mutants of cowpea varieties Gomati VU-89 and Pusa-578. The full-length gels are presented in Supplementary Fig. VII.

**Figure 11:**
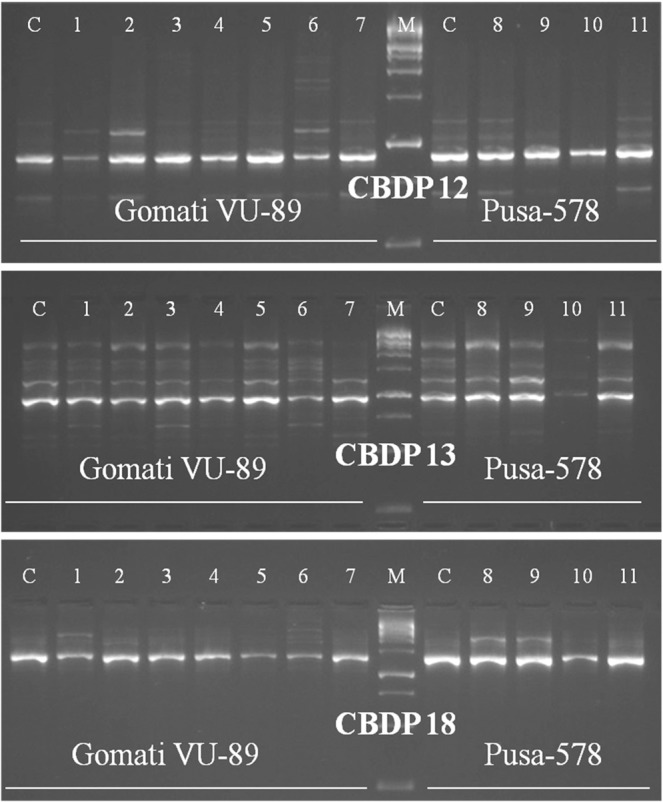
Cropped Gel profiles of markers generated using CAAT box derived primers. Picture a, b and c are showing markers generated using CBDP primers CBDP 12, 13 and 18 respectively in control and M_4_ high yielding mutants of cowpea varieties Gomati VU-89 and Pusa-578. The full-length gels are presented in Supplementary Figs. VIII, IX and X.

**Figure 12:**
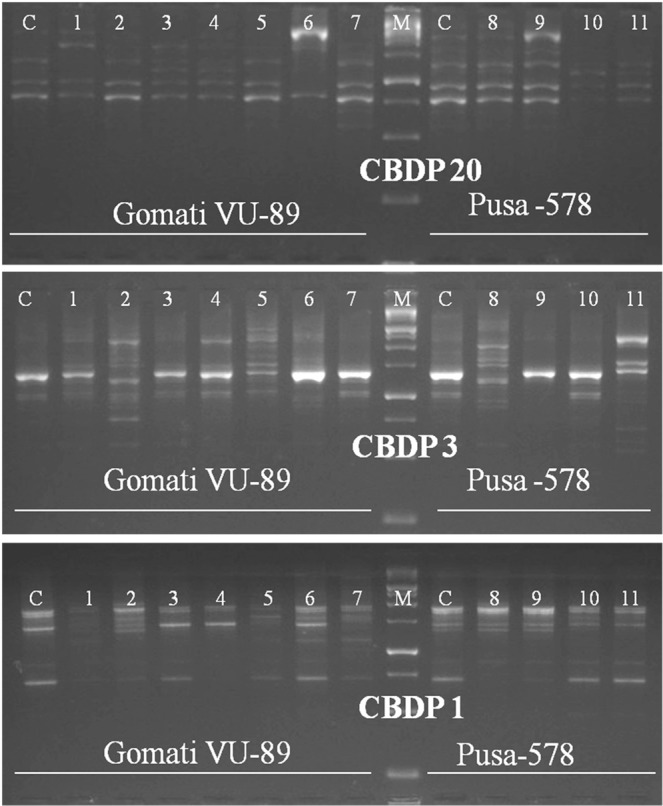
Cropped Gel profiles of markers generated using CAAT box derived primers. Picture a, b and c are showing markers generated using CBDP primers CBDP 20, 3 and 1 respectively in control and M_4_ high yielding mutants of cowpea varieties Gomati VU-89 and Pusa-578. The full-length gels are presented in Supplementary Figs. X and XI.

Of 25 CBDP primers tried, 20 generated polymorphic bands, while the remaining 5 primers produced monomorphic bands, the size of amplified DNA fragment ranged from 500 to 2000 bp in a mutagenized population of both the varieties. The selected 9 primers amplified 175 bands with 77 bands being polymorphic in the var. Gomati VU-89 and 121 bands with 59 bands being polymorphic in the var. Pusa-578 (Table 9; Figs. 10–12 and Supp. Figs. VII–XI). The maximum number of polymorphic amplified bands were 19 (CBDP 19) with an average of 8.56 in the var. Gomati VU-89 and 10 (CBDP 19) with an average of 6.56 in the var. Pusa-578. The overall numbers of polymorphic bands were higher in the var. Pusa-578 than in the var. Gomati VU-89. The values of polymorphism were between 73.07% (CBDP 19) and 29.41% (CBDP 17) with an average of 42.32 in the var. Gomati VU-89 and 71.42% (CBDP 19) and 36.84% (CBDP 20) with an average of 51.38 in the var. Pusa-578. The maximum PIC values were 0.85 (CBDP 20) with an average of 0.72 in the var. Gomati VU-89 and 0.86 (CBDP 20) with an average of 0.76 in the var. Pusa-578, respectively (Table 9). A particular CBDP primer acts as a forward and a reverse primer by annealing to promoters of any two genes and led to a generation of intervening genomic regions. Hence, any change in nucleotides, intervening genomic regions or disparity at primer binding site may result in the synthesis of variable length of fragments among the mutants which are genetically diverse from each other. The main benefit of employing the CBDP technique is that markers are gene promoter derived sequences that control important processes like gene expression, while other markers such as RAPD, AFLP, and ISSR are derived from random sequences of DNA. As a result, any estimation of CBDP marker based genetic diversity could reveal functional variation among the mutant population.

**Table 9:** Details of CBDP primers, GC content, total bands generated and polymorphic information for each primer.

		Gomati VU-89				Pusa-578			
Primer ID	GC %	TB	TPB	Polymorphism %	PIC	TB	TPB	Polymorphism %	PIC
CBDP-12	49.55	12	4	33.33	0.58	9	5	66.66	0.80
CBDP-13	50.00	23	7	30.43	0.55	16	6	37.50	0.80
CBDP-18	48.48	8	4	50.00	0.69	7	4	57.14	0.76
CBDP-17	44.56	17	5	29.41	0.75	7	3	42.85	0.70
CBDP-19	49.57	26	19	73.07	0.74	14	10	71.42	0.77
CBDP-16	50.23	15	5	33.33	0.75	14	7	50.00	0.83
CBDP-20	42.36	28	14	50.00	0.85	19	7	36.84	0.86
CBDP-3	46.59	19	7	36.84	0.76	14	8	57.14	0.80
CBDP-1	43.15	27	12	44.44	0.80	21	9	42.85	0.55
**Average**	—	19.44	8.56	42.32	0.72	13.44	6.56	51.38	0.76
**Total**	—	175	77	380.85	6.47	121	59	462.4	6.87

TB-Total number of bands, TPB-Total polymorphic bands, PIC-Polymorphic information content.

### Genetic divergent analysis

The SDS-PAGE based distance matrix and the Jaccard’s coefficient revealed a significant level of dissimilarity among the mutant population. The mutants viz., Gomati VU-89-A (0.575) and Pusa-578-A (0.238) were most divergent from the control population (Tables 10 and 11). On the basis of our results, we were able to differentiate mutants and to construct their dendrogram. The intensity graph and dendrogram revealed that substantial degree of variation in the expressed polypeptide among the mutants have been induced by the gamma rays and SA employed alone and in combination.

**Table 10:** Distance and Similarity Matrix based on Jaccard’s coefficient of SDS-PAGE profile of seven M_4_ high yielding mutants and control plants of Gomati VU-89.

		Gomati VU-89	Gomati VU-89-A	Gomati VU-89-B	Gomati VU-89-C	Gomati VU-89-D	Gomati VU-89-E	Gomati VU-89-F	Gomati VU-89-G	
Gomati VU-89	**DISTANCE**	**X**	0.575	0.368	0.235	0.333	0.571	0.400	0.400	**SIMILARITY**
Gomati VU-89-A		0.575	**X**	0.556	0.211	0.375	0.412	0.353	0.353	
Gomati VU-89-B		0.368	0.556	**X**	0.286	0.238	0.400	0.174	0.227	
Gomati VU-89-C		0.235	0.211	0.286	**X**	0.235	0.211	0.375	0.467	
Gomati VU-89-D		0.333	0.375	0.238	0.235	**X**	0.375	0.400	0.400	
Gomati VU-89-E		0.571	0.412	0.400	0.211	0.375	**X**	0.278	0.278	
Gomati VU-89-F		0.400	0.353	0.174	0.375	0.400	0.278	**X**	0.467	
Gomati VU-89-G		0.400	0.353	0.227	0.467	0.400	0.278	0.467	**X**

**Table 11:** Distance and Similarity Matrix based on Jaccard’s coefficient of SDS-PAGE profile of four M_4_ high yielding mutants and control plants of Pusa-578.

		Pusa-578	Pusa-578-A	Pusa-578-B	Pusa-578-C	Pusa-578-D	
Pusa-578	**DISTANCE**	**X**	0.238	0.038	0.080	0.174	**SIMILARITY**
Pusa-578-A		0.238	**X**	0.350	0.125	0.080	
Pusa-578-B		0.038	0.350	**X**	0.556	0.120	
Pusa-578-C		0.080	0.125	0.556	**X**	0.217	
Pusa-578-D		0.174	0.080	0.120	0.217	**X**	

**Figure 13:**
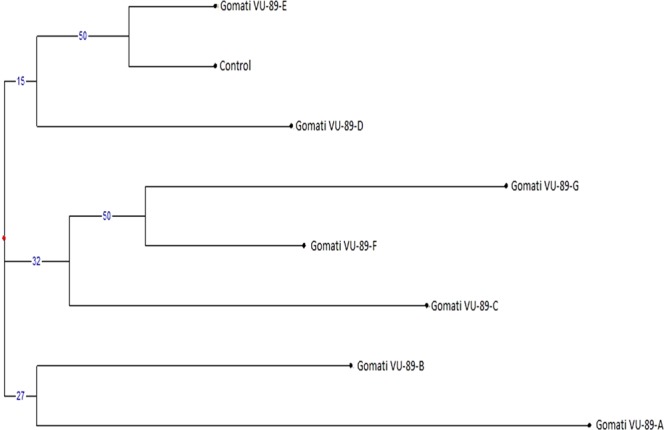
Dendrogram analysis of control and seven M_4_ high yielding mutants based on SSR profile of DNA sample of cowpea var. Gomati VU-89.

**Figure 14:**
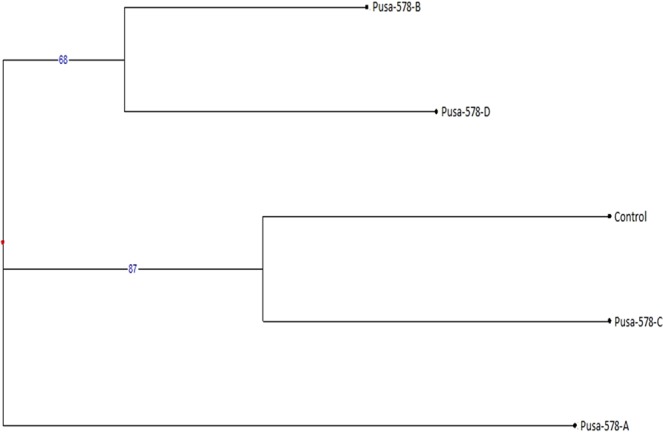
Dendrogram analysis of control and four M_4_ high yielding mutants based on SSR profile of DNA sample of cowpea var. Pusa-578.

The SSR based relative similarity and dissimilarity matrix of mutant and control population generated dendrograms are presented in Figs. 13 and 14. The genetic dissimilarity coefficient of the mutants and parental genotypes ranged from 0.200 (between Gomati VU-89-A and Gomati VU-89-B) to 1.000 (between Gomati VU-89 and Gomati VU-89-E) in the var. Gomati VU-89, while it ranged from 0.125 (between Pusa-578 and Pusa-578-A) to 0.385 (between Pusa-578 and Pusa-578-B) in the var. Pusa-578 (Tables 12 and 13). In the present study, Gomati VU-89-E (1.000) and Pusa-578-B (0.385) were recorded to be a genetically most divergent mutant from the control population of Gomati VU-89 and Pusa-578, respectively. This showed that the mutagen doses mutated the var. Gomati VU-89 relatively more than the var. Pusa-578. The dendrogram showed mutant lines of Gomati VU-89 were arranged in five clusters i.e., (I) Gomati VU-89-E and Control, (II) Gomati VU-89-D, (III) Gomati VU-89-G and Gomati VU-89-F, (IV) Gomati VU-89-C, (V) Gomati VU-89-B and Gomati VU-89-A (Fig. 13). In the var. Pusa-578 members were arranged in three clusters. A unique pattern was observed in both the dendrograms, i.e., (I) Pusa-578-B and Pusa-578-D, (II) Control and Pusa-578-C, (III) Pusa-578-A (Fig. 14). The dendrogram separated the control plants from that of mutant progenies and therefore, we can conclude that a considerable degree of genetic variability has been induced in the M_4_ high yielding mutants.

**Table 12:** Distance and Similarity Matrix based on Jaccard’s coefficient of SSR profile of seven M_4_ high yielding mutants and control plants of Gomati VU-89.

		Gomati VU-89	Gomati VU-89-A	Gomati VU-89-B	Gomati VU-89-C	Gomati VU-89-D	Gomati VU-89-E	Gomati VU-89-F	Gomati VU-89-G	
Gomati VU-89	**DISTANCE**	**X**	0.385	0.636	0.500	0.636	1.000	0.636	0.500	**SIMILARITY**
Gomati VU-89-A		0.385	**X**	0.200	0.286	0.385	0.385	0.286	0.286	
Gomati VU-89-B		0.636	0.200	**X**	0.286	0.500	0.636	0.500	0.286	
Gomati VU-89-C		0.500	0.286	0.286	**X**	0.385	0.500	0.500	0.385	
Gomati VU-89-D		0.636	0.385	0.500	0.385	**X**	0.636	0.636	0.385	
Gomati VU-89-E		1.000	0.385	0.636	0.500	0.636	**X**	0.636	0.500	
Gomati VU-89-F		0.636	0.286	0.500	0.500	0.636	0.636	**X**	0.500	
Gomati VU-89-G		0.500	0.286	0.286	0.385	0.385	0.500	0.500	**X**

**Table 13:** Distance and Similarity Matrix based on Jaccard’s coefficient of SSR profile of four M_4_ high yielding mutants and control plants of Pusa-578.

		Pusa-578	Pusa-578-A	Pusa-578-B	Pusa-578-C	Pusa-578-D	
Pusa-578	**DISTANCE**	**X**	0.125	0.385	0.200	0.125	**SIMILARITY**
Pusa-578-A		0.125	**X**	0.200	0.125	0.215	
Pusa-578-B		0.385	0.200	**X**	0.200	0.125	
Pusa-578-C		0.200	0.125	0.200	**X**	0.385	
Pusa-578-D		0.125	0.215	0.125	0.385	**X**	

**Figure 15:**
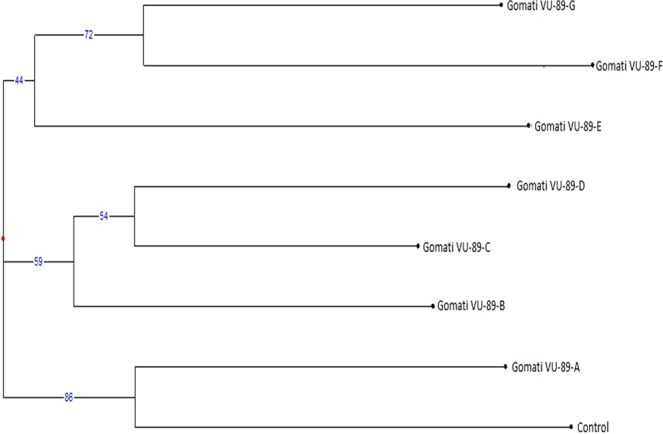
Dendrogram analysis of control and seven M_4_ high yielding mutants based on CBDP profile of DNA sample of cowpea var. Gomati VU-89.

**Figure 16:**
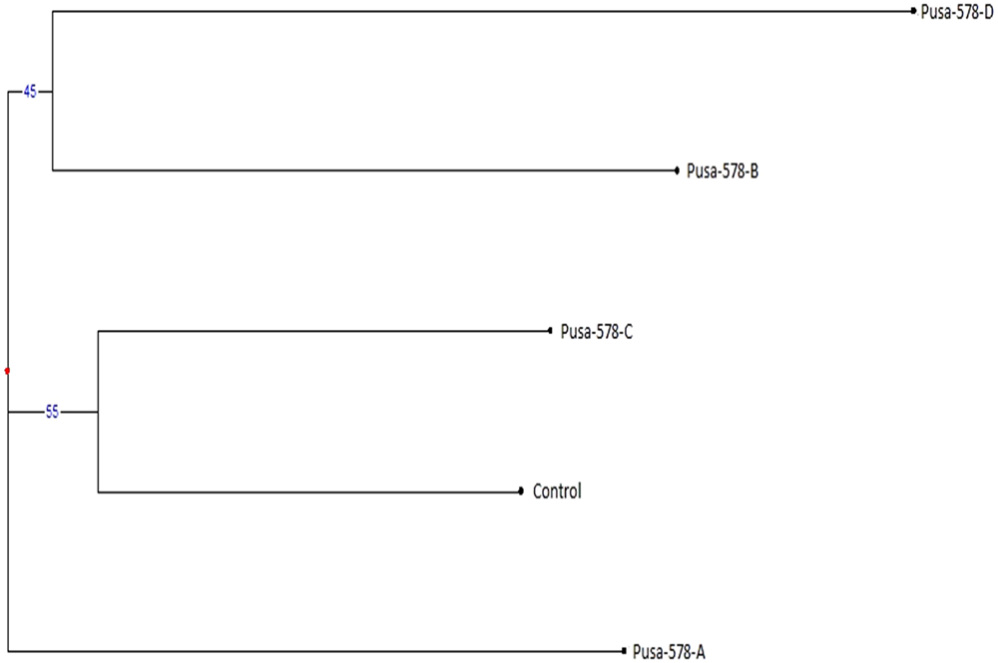
Dendrogram analysis of control and four M_4_ high yielding mutants based on CBDP profile of DNA sample of cowpea var. Pusa-578.

The CBDP based genetic dissimilarity coefficient of the mutants and controls ranged from 0.172 (between Gomati VU-89 and Gomati VU-89-F) to 0.524 (between Gomati VU-89-C and Gomati VU-89-D) in the var. Gomati VU-89, while it ranged from 0.111 (between Pusa-578-A and Pusa-578-D) to 0.500 (between Pusa-578 and Pusa-578-C) in the var. Pusa-578 (Tables 14 and 15). The dendrogram indicated a high level of genetic variation and differentiated mutant lines into three clusters each of which was represented by a separate mutant line. The bootstrap results showed that grouping of genotypes was dependable at major nodes (Figs. 15, 16). Similar Jaccard’s similarity coefficient based clustering patterns for mutant cultivars of lentil using RAPD have been reported by Laskar *et al*.^27^.

**Table 14:** Distance and Similarity Matrix based on Jaccard’s coefficient of CBDP profile of seven M_4_ high yielding mutants and control plants of Gomati VU-89.

		Gomati VU-89	Gomati VU-89-A	Gomati VU-89-B	Gomati VU-89-C	Gomati VU-89-D	Gomati VU-89-E	Gomati VU-89-F	Gomati VU-89-G	
Gomati VU-89	**DISTANCE**	**X**	0.417	0.241	0.360	0.231	0.188	0.172	0.214	**SIMILARITY**
Gomati VU-89-A		0.417	**X**	0.333	0.360	0.231	0.267	0.259	0.259	
Gomati VU-89-B		0.241	0.333	**X**	0.500	0.417	0.333	0.241	0.333	
Gomati VU-89-C		0.360	0.360	0.500	**X**	0.524	0.310	0.214	0.308	
Gomati VU-89-D		0.231	0.231	0.417	0.524	**X**	0.241	0.280	0.280	
Gomati VU-89-E		0.188	0.267	0.333	0.310	0.241	**X**	0.188	0.357	
Gomati VU-89-F		0.172	0.259	0.241	0.214	0.280	0.188	**X**	0.417	
Gomati VU-89-G		0.214	0.259	0.333	0.308	0.280	0.357	0.417	**X**

**Table 15:** Distance and Similarity Matrix based on Jaccard’s coefficient of CBDP profile of four M_4_ high yielding mutants and control plants of Pusa-578.

		Pusa-578	Pusa-578-A	Pusa-578-B	Pusa-578-C	Pusa-578-D	
Pusa-578	**DISTANCE**	**X**	0.407	0.286	0.500	0.176	**SIMILARITY**
Pusa-578-A		0.407	**X**	0.286	0.286	0.111	
Pusa-578-B		0.286	0.286	**X**	0.308	0.152	
Pusa-578-C		0.500	0.286	0.308	**X**	0.226	
Pusa-578-D		0.176	0.111	0.152	0.226	**X**

Apart from the little differences both the markers resulted into a similar pattern of clustering and creation of dendrograms like Gomati VU-89-F and Gomati VU-89-G were always grouped together and similarly, Pusa-578-B and Pusa-578-D were also grouped together in both the marker analysis. The dendrogram showed control and mutant plants in separate clusters, which reconfirmed that considerable degree of genetic variability was generated by gamma rays and SA treatments used alone or in combination. Hence, the genetically diverged M_4_ mutants developed can be employed as mutant varieties directly or as parents in crossbreeding programmes for advanced improvement and fixation of the traits in cowpea.

## Conclusions

The current research led to the development of high yielding cowpea mutant lines and the seeds of mutant lines are stored in the department seed bank for further farmer’s field based characterization and dissemination to the cowpea breeders. A good degree of genetic variability has been induced in the mutant lines as revealed by the manifold increase in GCV, h^2^ and GA. Assessments on NRA, chlorophyll, carotenoids, protein and mineral elements showed improved physiological and biochemical activity in the high yielding mutant lines of cowpea. SDS-PAGE analysis revealed the expression of new polymorphic protein bands among the selected high yielding mutant lines. CBDP and SSR markers have generated a considerable amount of polymorphism which in turn validates the genetic divergence of mutants from the control population. Cluster analysis separated the control plants of both the cowpea varieties into separate clusters evidently confirmed that the employed mutagens successfully created heritable genetic changes with considerable genetic gains in the M_4_ high yielding mutants compared to their respective controls. The genetic divergent analysis using molecular markers confirmed that the selected M_4_ high yielding mutants, especially Gomati VU-89-G and Pusa-578-C, induced in the present study contains novel combination of genes and has significant breeding implications in the development of new high yielding cowpea varieties, either directly or as parents in cross breeding programmes.

## Supplementary information

Supplementary Info.
