# Supplementary material for: Characterization of Induced High Yielding Cowpea Mutant Lines Using Physiological, Biochemical and Molecular Markers
Source: Sci Rep. 2020 Feb 28;10:3687. doi: 10.1038/s41598-020-60601-6 (PMC7048850; doi:10.1038/s41598-020-60601-6)
Supplement: Supplementary file 1 — Supplementary Info. [file 41598_2020_60601_MOESM1_ESM.pdf]

Title: Characterization of Induced High Yielding Cowpea Mutant Lines Using Physiological, Biochemical and Molecular Markers

Aamir Raina[1,2*], Rafiul Amin Laskar[1**], Younas Rasheed Tantray[3], Shahnawaz Khursheed[1], Mohd. Rafiq Wani[1,4], Samiullah Khan[1]
[1]Mutation Breeding Laboratory, Department of Botany, Aligarh Muslim University, Aligarh, India
[2]Botany Section, Women's College, Aligarh Muslim University, Aligarh, India
[3]Department of Botany, Punjabi University, Patiala, India
[4]Department of Botany, Abdul Ahad Azad Memorial Government Degree College, Srinagar, Jammu and Kashmir, India
Corresponding author: *aamir854@gmail.com; **rafihkd@gmail.com

Supplementary Table 1: Isolation of mutant lines at different doses of gamma rays and sodium azide.

Supplementary Table 2: Primer information of genic-SSR markers.

Supplementary Table 3: Primer information of CBDP markers.

Supplementary Fig. 1A: Field picture showing different treated population sown in RCBD manner: C=Control, G1=100 Gy gamma rays, G2=200 Gy gamma rays, G3=300 Gy gamma rays, G4=400 Gy gamma rays, S1=0.01% SA, S2=0.02% SA, S3=0.03% SA, S4=0.04% SA, G1+S1=100 Gy gamma rays+0.01% SA, G2+S2=200 Gy gamma rays+0.02% SA, G3+S3=300 Gy gamma rays+0.03% SA, G4+S4=400 Gy gamma rays+0.04% SA.

Supplementary Fig. 1B: a- Experimental field; b-e-High yield mutant lines.

Supplementary Figure II. The full length gel profiles of markers generated using SSR primers VU-1, VU-2, VU-27 and VU-19 respectively in control and M_4 high yielding mutants of cowpea varieties Gomati VU-89 and Pusa-578.

Supplementary Figure III. The full length gel profiles of markers generated using SSR primers VU-10, VU-17, VU-42 and VU-47 respectively in control and M_4 high yielding mutants of cowpea varieties Gomati VU-89 and Pusa-578.

Supplementary Figure IV. The full length gel profiles of markers generated using SSR primers MB-61, MB-12 and MB-25 respectively in control and M_4 high yielding mutants of cowpea varieties Gomati VU-89 and Pusa-578.

Supplementary Figure V. The full length gel profiles of markers generated using SSR primers MB-31, MB-26 and MB-38 respectively in control and M_4 high yielding mutants of cowpea varieties Gomati VU-89 and Pusa-578.

Supplementary Figure VI. The full length gel profiles of markers generated using SSR primers MB-13, VU-23, VU-25 and MB-36 respectively in control and M_4 high yielding mutants of cowpea varieties Gomati VU-89 and Pusa-578.

Supplementary Figure VII. The full length gel profiles of markers generated using CAAT primers CBDP-17, CBDP-19 and CBDP-16 respectively in control and M_4 high yielding mutants of cowpea varieties Gomati VU-89 and Pusa-578.

Supplementary Figure VIII. The full length gel profiles of markers generated using CAAT primers CBDP-12, CBDP-10 and CBDP-25 respectively in control and M_4 high yielding mutants of cowpea varieties Gomati VU-89 and Pusa-578.

Supplementary Figure IX. The full length gel profiles of markers generated using CAAT primers CBDP-13, and CBDP-8 respectively in control and M_4 high yielding mutants of cowpea varieties Gomati VU-89 and Pusa-578.

Supplementary Figure X. The full length gel profiles of markers generated using CAAT primers CBDP-18, CBDP-20 and CBDP-7 respectively in control and M_4 high yielding mutants of cowpea varieties Gomati VU-89 and Pusa-578.

Supplementary Figure XI. The full length gel profiles of markers generated using CAAT primers CBDP-1, CBDP-3 and CBDP-6 respectively in control and M_4 high yielding mutants of cowpea varieties Gomati VU-89 and Pusa-578.

Supplementary Table 1: Isolation of mutant lines at different doses of gamma rays and sodium azide.

STRAIN NUMBER	ORIGIN/ TREATMENT	Remarks
**Gomati VU-89**	**Gomati VU-89 (Control)**	
Gomati VU-89-A	0.02% SA	High yield
Gomati VU-89-B	100Gy γ rays+0.01% SA	High yield
Gomati VU-89-C	200 Gy γ rays	High yield
Gomati VU-89-D	200Gy γ rays+0.02% SA	High yield
Gomati VU-89-E	100Gy γ rays	High yield
Gomati VU-89-F	200Gy+0.02% SA	High yield
Gomati VU-89-G	100Gy γ rays	High yield
**Pusa-578**	**Pusa-578 (Control)**	
Pusa 578-A	0.02% SA	High yield
Pusa 578-B	200Gy γ rays + 0.02% SA	High yield
Pusa 578-C	200Gy γ rays	High yield
Pusa 578-D	100 Gy γ rays +0.01% SA	High yield

Supplementary Table 2: Primer information of genic-SSR markers.

NAME	Primer sequence (forward)	Primer sequence (reverse)
VU-1	AACAAGATGTGGCATGCTGA	TGAAAACGGAAAAGGGATCA
VU-2	GAAACTAGCACCAAATCCAACA	GAGCAAAAGCCTCCATCACT
VU-3	GCACCCAATCAAACACACAC	GAAGCGGATTTGAGAGTTGG
VU-8	TCAAAAACACAGGTCCTCCA	CATCCCGTGAAATTCAACAA
VU-10	TCAAAACTTCAACCCAGACA	AAAAAGGAAGTCCATTGCTC
VU-17	GGATATCATAGCAAGTCGAA	AAGGAGTGCATCCTAAACTC
VU-19	AGAACCCAGCATACCTGCAT	CCTCGCCAATGATTCTGAG
VU-23	CGTACCTAATGTGAAGGTTCGTT	AAGGCAAAAAGCTCTTGCAG
VU-25	AGGGATGAGTTCCTTCAACG	AAGAAGTGGTGAGGGCACAG
VU-27	TCCATCCACCATTTTCCATC	ATGGGAATGCCCGAGAGT
VU-29	TTTTTCTCGACACACGGTGA	TTTCCCCCTCTCTCACACAC
VU-42	ATGTTATACGCCGGCAAAGT	TCTGGGTGCTTTGGAAAATC

VU-47	TGTTTTTCGCTATGCCTCAA	GGCAGCTAGATTCGTCCTTG
VU-53	CATTCCACGATACATACATACCC	CGTGAAAGGATCTGAATTGG
VU-61	ACCCAACCCTTCTCATAGGG	CAACACTGCTCGATCCTCCT
VU-62	TTGTCAAAAATCAGAAGCTC	CTTGCATTCACTTCTACCAC
VU-94	CAACCCCATCACTGTGTCAG	GATGCAAGGAGAAGGAGCAG
VU-95	CCCAAGAGATTTGCTCTGAA	AAACAACAGGGAATTTGCAG
MB-1	AGAAGGCCTGCAAGTGGAAA	TCTCTGCAATGCACAAGTCT
MB-2	TGGCAAACCCCATATTTACCA	AGGAAGCAAGTAGGGGGTTTG
MB-3	AAGAACTCCGCCGGAAGTAC	TCTTGCAGCGTTCCTGATGT
MB-4	TGCCCTCTGTTATGGTGGAG	AGTTGCATCGGCCTCATCTC
MB-5	AGCACCCCAGCTTGAAGAAA	TGCTTTGCGGCAAATTTCCA
MB-6	GCAGAAGGAAGCTCAAGATCG	GCTTCCCACAACTCCCAGAA
MB-7	AACGACTGCAATGCACAACC	GAGTTGTCTGAGGCTGAGGG
MB-8	TCGGTGTGTCGTTGATTGGA	CAGCAATTGAAACATTTGTTCGA
MB-9	TGCCTCATGAGGGAAATCAACA	ACACACACTTACTGCGAAGT
MB-10	AGGAGCCAACAACAAACCCA	AGCAACGCCATCATCATCCA
MB-11	AGAACCATGCCACGTGACAT	GTCCAACCACGCAAACTCAC
MB-12	GATCGGCCTCATGCTCCTTT	GTGGTGGTGAGAGTGGGAAG
MB-13	AGATCCCATGCTCGCTTCTG	CAGCTTGCTTTCCCTTGCTG
MB-14	GGCCCCAGTGAATACTATCTCC	AGGTAAACCAATAAACCATTTTCCT
MB-15	GGCACACTCATGGACCTTCA	ATACCACTCTGAGCAACGGC
MB-16	AACGCCATAGGATTCATTCGA	AGCGTGAACGATCTTATTATTACCA
MB-17	CGCAAATGGAAACCCACCAG	TTATGCCACGAGCTTCGAGC
MB-18	ACGTGTAGAGAGACCACCGA	CCAAGCAGCAGAACCAACAC
MB-19	TGGACCTTTTTCCCCATTCATCT	AGTGCAGGCAGGAGCAAATA
MB-20	AAGCAAGATGACACGGAGCA	ATGGTTGAAGAAGCGGGAGG
MB-21	ATTGCCACCCCCATTTCCAT	AGCAGTCCACCACTCTCTCT
MB-22	GCCATCACCAACTACCCCTC	AGGGGAGGGCGTAGATGTAG
MB-23	AAGGAGGGATTCTCGCCTCT	TGGTACCCGAACTTCTTGGC
MB-24	AAACAGTCAGAGAGGTTTGCA	AAGCCACGACGATGAGGTTT
MB-25	ATGTCTGCATCATGGGAGCC	CCCCAACAACAGCAGCAATC
MB-26	ACCCTATGTGCAGTGCAACA	TCTCCTCCCTTGAGAGAGGC
MB-27	ATCAACCATGGCTGCCTCAT	GCTGACTGGAAGAAGAGCGT
MB-28	ACGAAATCAACGAGGCATATGA	ACTTTTGTTGCGGAGGGGAA
MB-29	AGTAGAGAACGCTCGGAGGT	TCCAGTGTAACTACCGCCAC
MB-30	AAGCGTGGAAGTGGAGTGAG	ACCGACTTAACGTTATTGAAAAGAGG
MB-31	GGGTGGGGAAAGGGAAGAAG	ACGCATACACACGCAGAGAA
MB-32	AAGACGACTGGACGATTCCG	CCGTCTTCGTGTTTCCTCCA
MB-33	TGGTCGGTGTGTTAGTGTCG	TGCATGGTGAAACCTTATCGT

MB-34	CTCCTGAGGGCACTGAACTG	GCTTCTGCAACGAGTTTCAACT
MB-35	CTCAACAAGTTCCTCAGCGC	CCAGAACCGGTGGAAGTCTC
MB-36	CCCAACCTCTCCGCAAAGAT	ACAGCCAATCCACGTACCTC
MB-37	ATCCTTCGTGGTCTCCGAGA	GGGGTTACAGGACCAGAAGC
MB-38	CCTCCCTCTCCCTCTTCCTC	TGGGATTCCGGGCAAATCTT
MB-39	CAGATTCCAACCCGAAGCCA	GCGAAAGAAGCTCGTCCTCT
MB-40	AAGAGAAGGGGTTCGTGCTC	AGGGCGAGAGAATCAAACCG
MB-41	AAGAAGGAAGCTCCAAGGCC	GCAGCCACGTGTTGCATTAT
MB-42	AAGAAAGGTGGAGGAGCGTG	ACCTCAACTTCTTCCATTTTTCAACT
MB-43	GCTTAACTTCAACGGCACCC	TTAGCCCTTCCGTTTCGCTT
MB-44	CTCCTGGGCACATTTCCACT	ACCACCCACATCATTTCCCC
MB-45	ACAGGCATTATGATACTTTCTGCA	GAAACTGCACAGCACTTGGT
MB-46	TTGTGGTGTCCGTAGTGAGC	TCGCTTCGGAAAGTGCTTCT
MB-47	ACCAAGAACAGAGCCAGAGC	GTTTTCCACTCCGATGCGTG
MB-48	GCAAATTGCAGCTCACATCG	TCTCGGAAGATTGGCTGGTT
MB-49	CCTTGCTCTTGTGTGCCTCT	CTCCACAGCATTGACCCCAT
MB-50	ACTGAGTCTCACCAGAGCCA	ATTCTCCGGCACTCAACAGG
MB-51	TCCGTGTTCTTAGCCAAGATCA	CTTGGCTGCAGCTGAAGTTC
MB-52	ACGCGTTGGGAAGGTTAAGA	GAAATTCCGATGCCCATGCC
MB-53	TGGCATTCTCCCAATTCCCT	TCCTCCTGATTGGACCTCTCA
MB-54	ACAGTGCTCCACAACAAGGA	GCGCTGATAGAGGCAGACTT
MB-55	CCTGCCACCCATCGACTTTA	GCAGTGGAGAAATGGTGACC
MB-56	CCCTCACAAACTCGAGACCC	GAAACGAAGGTGGCTGAGGA
MB-57	GCCATTGTAGAAACCCCAGC	ATATTGGGTGCTGCCGGTAC
MB-58	ATTTCCCTGTGCGCCCATAA	TCTGTTATGCAGCAGGCTCC
MB-59	AGAGGCCATGGTAGAAGGGT	AGCCTTGGAGCTGGTTGATG
MB-60	CCGGCATGTTAAGGTGGTCT	GAAGGTCATGACGAGGCCAA
MB-61	AGGCTGATTACAATGGCAAGGA	GGATCTGCACACACACATGC
MB-62	AGCAGGTTTCTTCTCACCGG	AGGAATTAGGGTTTGGGCGG
MB-63	GTCCGGATTGAAGGGTCACA	TATCCGAGAATCACCCCCGT
MB-64	CATGGGTCATGCACTTTCGT	CGCATCCATTGAAGACCAAGC
MB-65	AACCTTCCAGGAATGCCAGG	TTGCTTGACCTCTGCCTCAG
MB-66	AGTTAACACTCACCCTCGCG	TCAGCTTCGGAGTCGCATTT

Supplementary Table 3: Primer information of CBDP markers.

Primer ID	Primer Sequence[a]
CAAT1	TGAGCACGAT**CCAAT** AGC
CAAT2	TGAGCACGAT**CCAAT**AAT
CAAT3	TGAGCACGAT**CCAAT** ACC
CAAT4	TGAGCACGAT**CCAAT** AAG
CAAT5	TGAGCACGAT**CCAAT** CTA
CAAT6	TGAGCACGAT**CCAAT** CAG
CAAT7	TGAGCACGAT**CCAAT** CGA
CAAT8	TGAGCACGAT**CCAAT** CGG
CAAT9	TGAGCACGAT**CCAAT** GAT
CAAT10	TGAGCACGAT**CCAAT** GTT
CAAT11	TGAGCACGAT**CCAAT** TGC
CAAT12	TGAGCACGAT**CCAAT**ATA
CAAT13	TGAGCACGAT**CCAAT**GAG
CAAT14	TGAGCACGAT**CCAAT**GCG
CAAT15	TGAGCACGAT**CCAAT**TGA
CAAT16	TGAGCACGAT**CCAAT**TCA
CAAT17	TGAGCACGAT**CCAAT**TTG
CAAT18	CTGAGCACGAT**CCAAT**AG
CAAT19	CTGAGCACGAT**CCAAT**AC
CAAT20	CTGAGCACGAT**CCAAT**AT
CAAT21	CTGAGCACGAT**CCAAT**CA
CAAT22	CTGAGCACGAT**CCAAT**CG
CAAT23	CTGAGCACGAT**CCAAT**GG
CAAT24	CTGAGCACGAT**CCAAT**GA
CAAT25	CTGAGCACGAT**CCAAT**GT

Appendix I: Reagents used for the estimation of NRA, chlorophyll and carotenoid contents and mineral elements.

(A) 0.1M phosphate buffer (7.4 pH)

27.2 g of KH2PO4 and 45.63 g of K2HPO4.7H2O were dissolved separately in 1000 ml of DDW.

The above solution of KH2PO4 and K2HPO4.7H2O were mixed in the ratio of 36:64, respectively.

(B) 0.2M potassium nitrate

20.2 g of KNO3 was dissolved in sufficient DDW and final volume was made upto 1000 ml using DDW

(C) Isopropanol (5%)

5 ml of isopropanol was pipette into sufficient DDW and final volume was made upto 100 ml, using DDW.

(D) Sulphanilamide (1%)

1 g of sulphamnilamide was dissolved in 100 ml of 3N HCL.

3N HCL was prepared by dissolving 25.86 ml of HCl in sufficient DDW and final volume was maintained to 100 ml, using DDW.

(E) N-1-nehthyl-ethylenediamine dihydro chloride-HCl (NED-HCl) (0.02%)

20 mg of NED-HCl was dissolved in sufficient DDW and final volume was made upto 100 ml, using DDW.

(F) Acetone (80%)

80% acetone was prepared bu mixing 80 ml of acetone with 20 ml of DDW.

Appendix II: Reagents and components used in SDS-PAGE.

(A) Separating gel: (10%) (for 5 ml)

i. 2 ml ddH_2O
ii. 1.67 ml 30% acrylamide/Bis
iii. 1.25 ml 1.5 M Tris (pH 8.8)
iv. 25 µl 20% SDS
v. 25 µl 10% ammonium persulphate
vi. 2.5 µl TEMED

(B) Stacking gel: (5 %) (for 3 ml)

vii. 2.088 ml dH_2O
viii. 0.506 ml 30% acrylamide/Bis
ix. 0.375 ml 1 M Tris (pH 6.8)
x. 15 µl 20% (w/v) SDS
xi. 15 µl 10% ammonium persulphate
xii. 1.5 µl TEMED

(C) SDS protein sample buffer: (2X)

xiii. 1.25 ml 1 M Tris HCl (pH 6.8)
xiv. 4.0 ml 10% (w/v) SDS
xv. 2.0 ml glycerol
xvi. 0.5 ml 0.5 M EDTA
xvii. 4 mg bromophenol blue
xviii. 0.2 ml 14.3 M β-mercaptoethanol
xix. Bring the volume to 10 ml by adding ddH_2O

(D) Running buffer: (10X)

xx. 30.30 g Tris-base
xxi. 144.0 g glycine
xxii. 10.0 g SDS
xxiii. Completely dissolve it in 800 ml ddH_2O and then bring the final volume to 1000 ml by adding more ddH_2O

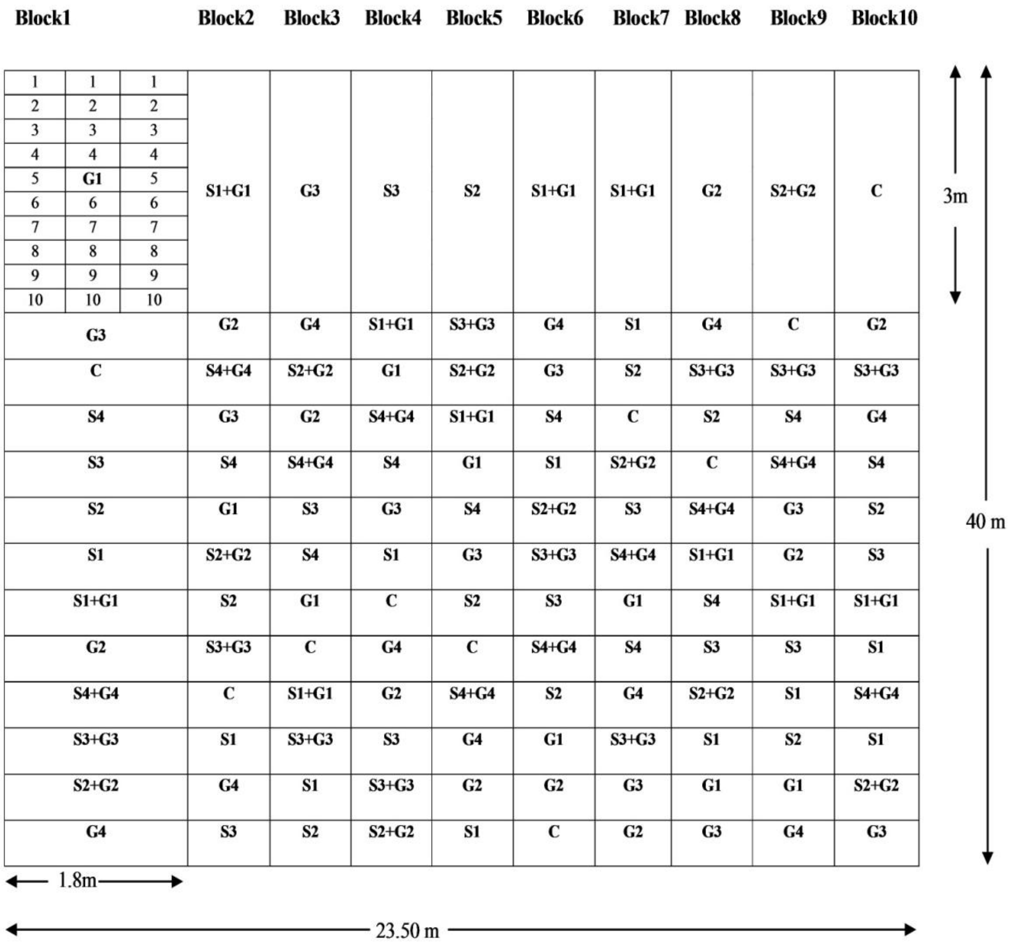

Supplementary Fig. 1A: Field picture showing different treated population sown in RCBD manner: C=Control, G1=100 Gy gamma rays, G2=200 Gy gamma rays, G3=300 Gy gamma rays, G4=400 Gy gamma rays, S1=0.01% SA, S2=0.02% SA, S3=0.03% SA, S4=0.04% SA, G1+S1=100 Gy gamma rays+0.01% SA, G2+S2=200 Gy gamma rays+0.02% SA, G3+S3=300 Gy gamma rays+0.03% SA, G4+S4=400 Gy gamma rays+0.04% SA.

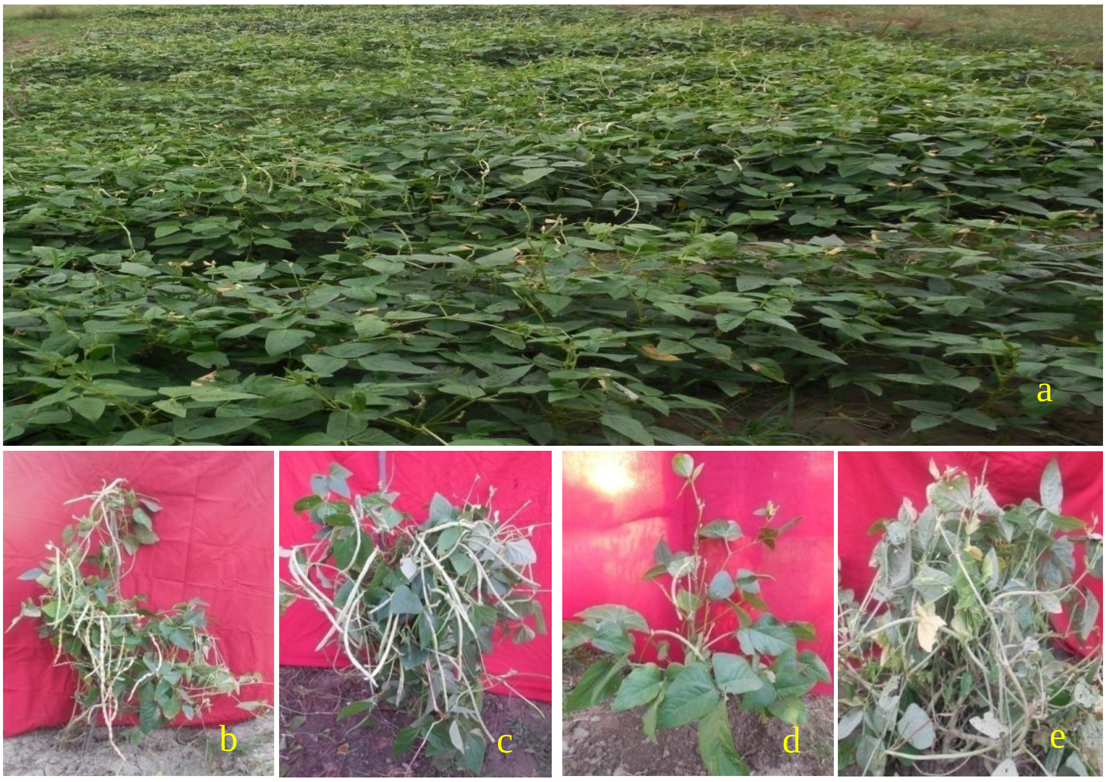

Supplementary Fig. 1B: a- Experimental field; b-e-High yield mutant lines.

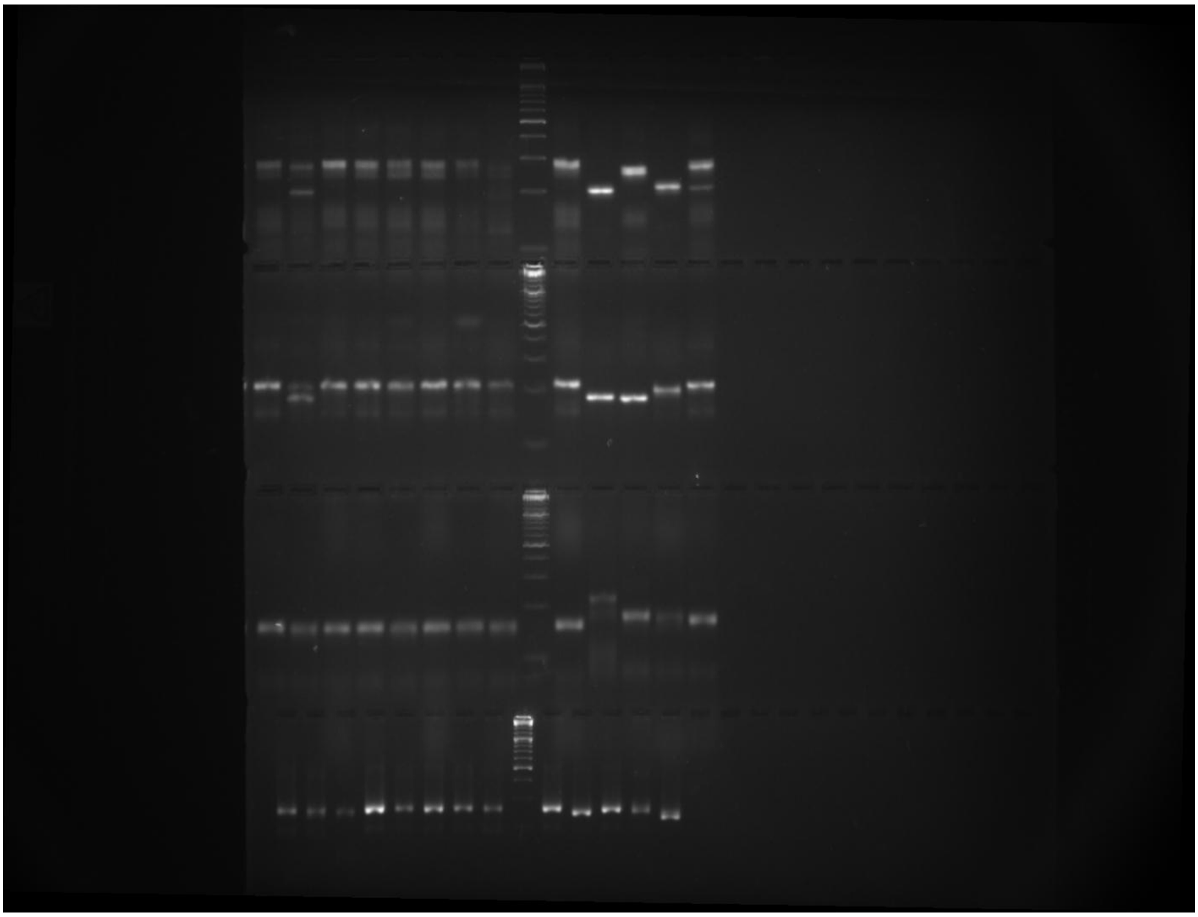

Supplementary Figure II. The full length gel profiles of markers generated using SSR primers VU-1, VU-2, VU-27 and VU-19 respectively in control and M_4 high yielding mutants of cowpea varieties Gomati VU-89 and Pusa-578.

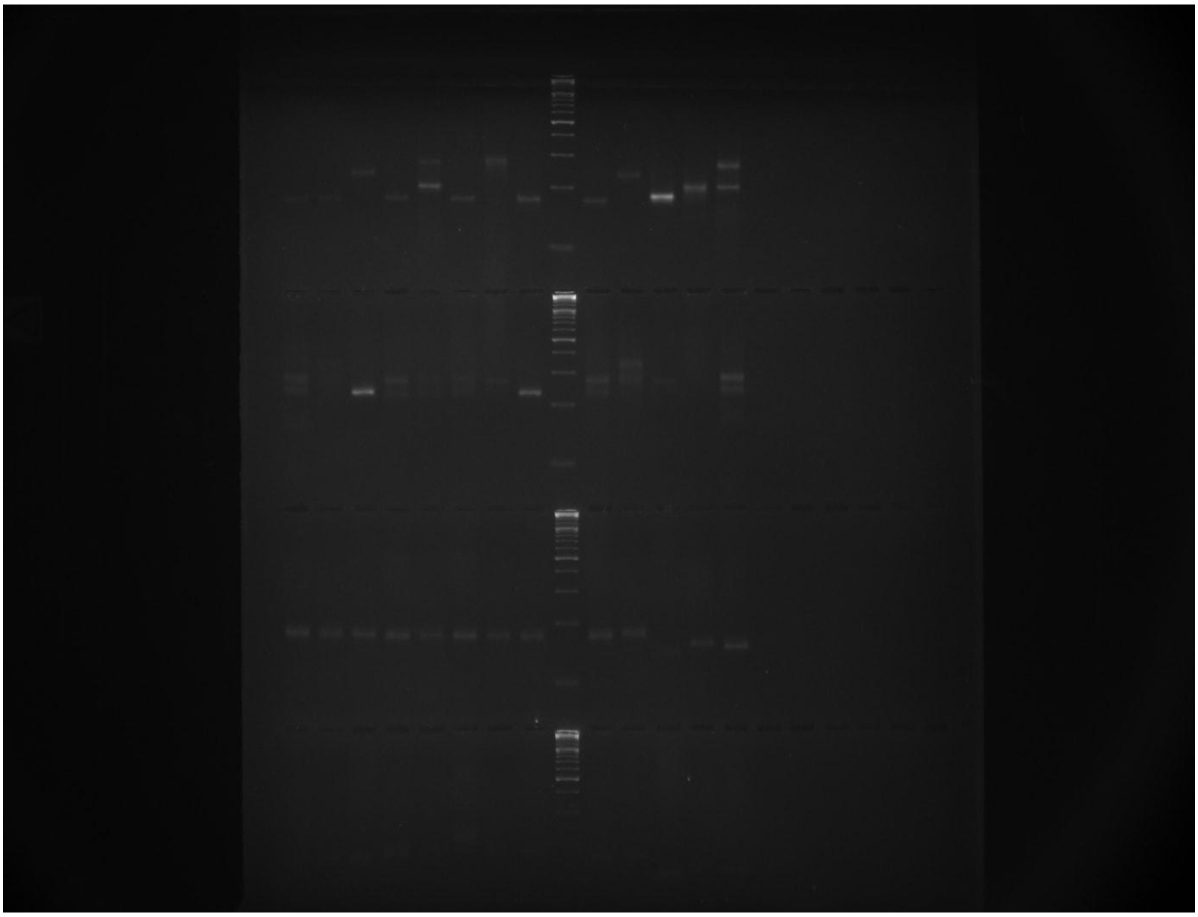

Supplementary Figure III. The full length gel profiles of markers generated using SSR primers VU-10, VU-17, VU-42 and VU-47 respectively in control and M_4 high yielding mutants of cowpea varieties Gomati VU-89 and Pusa-578.

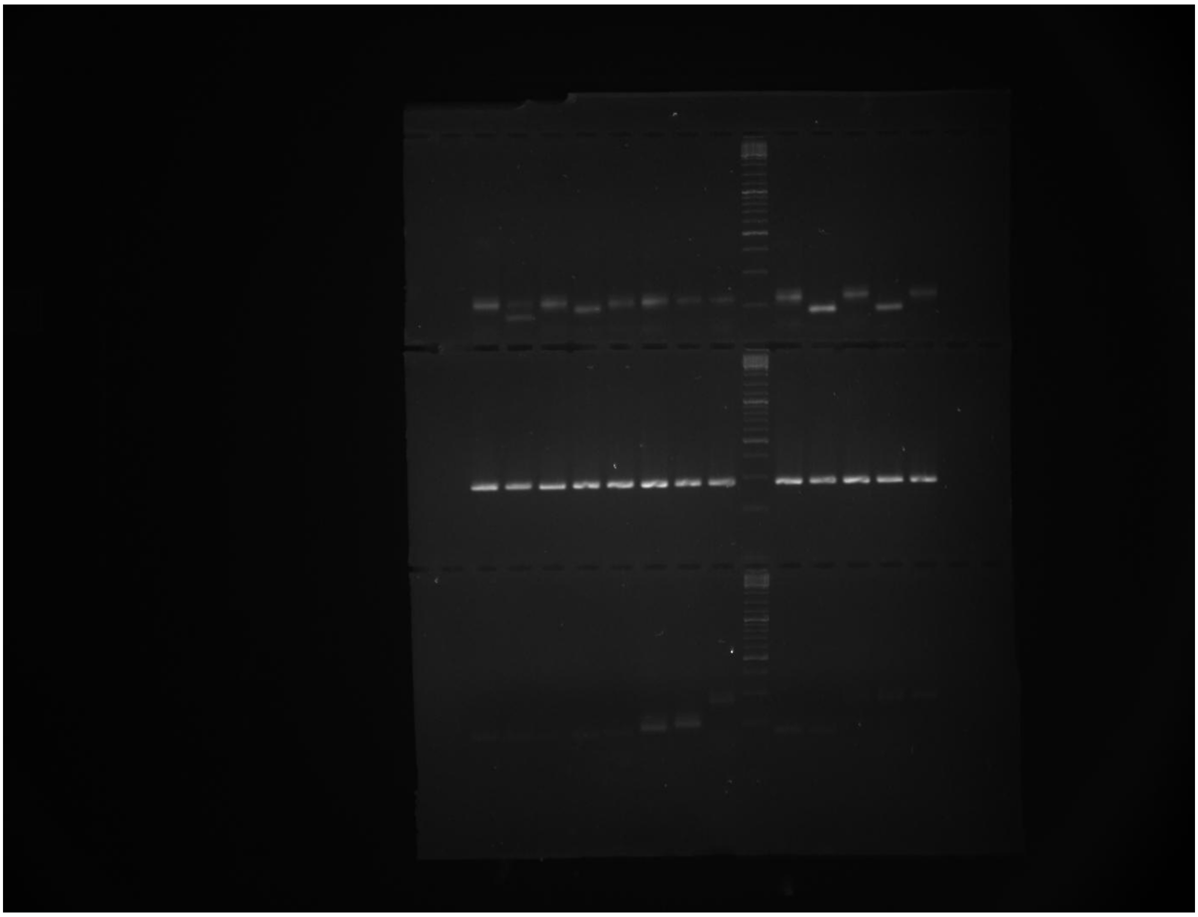

Supplementary Figure IV. The full length gel profiles of markers generated using SSR primers MB-61, MB-12 and MB-25 respectively in control and M_4 high yielding mutants of cowpea varieties Gomati VU-89 and Pusa-578.

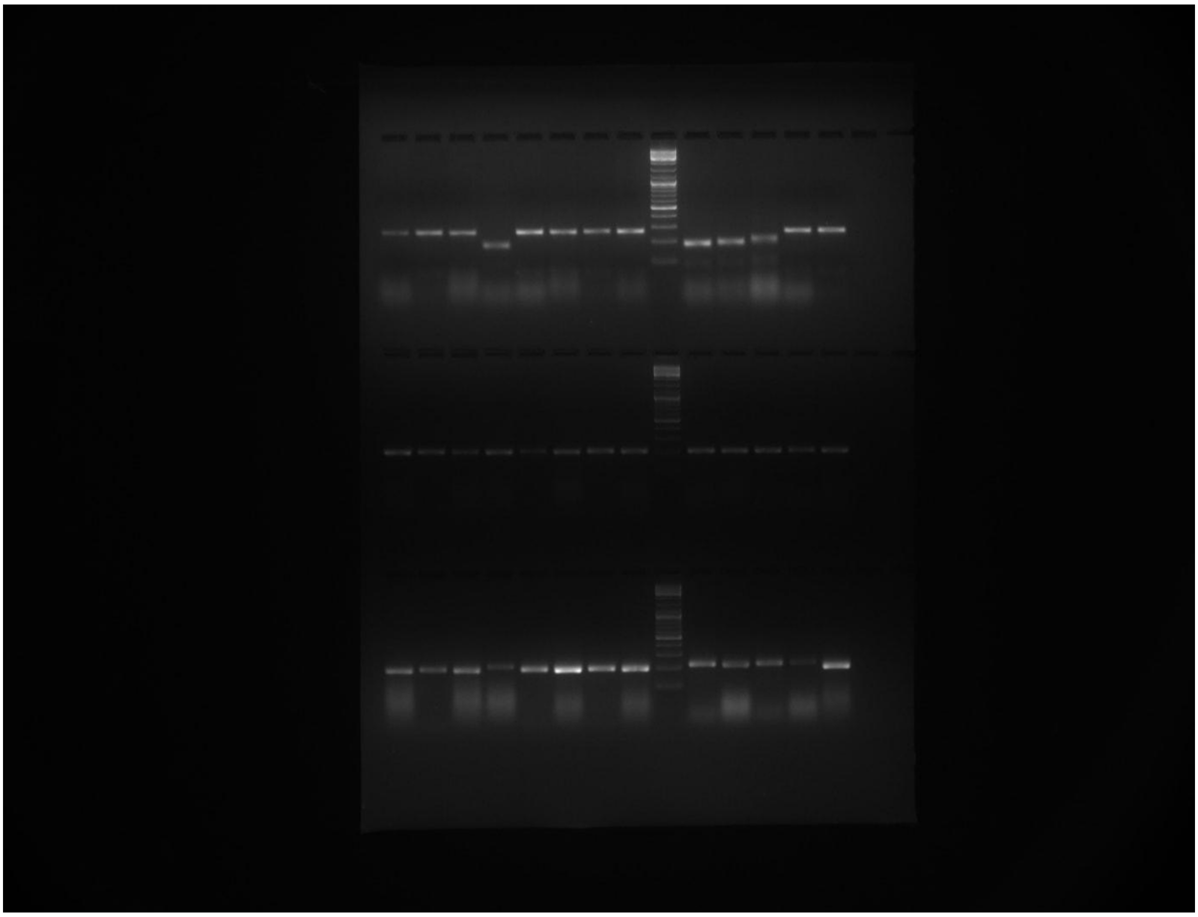

Supplementary Figure V. The full length gel profiles of markers generated using SSR primers MB-31, MB-26 and MB-38 respectively in control and M_4 high yielding mutants of cowpea varieties Gomati VU-89 and Pusa-578.

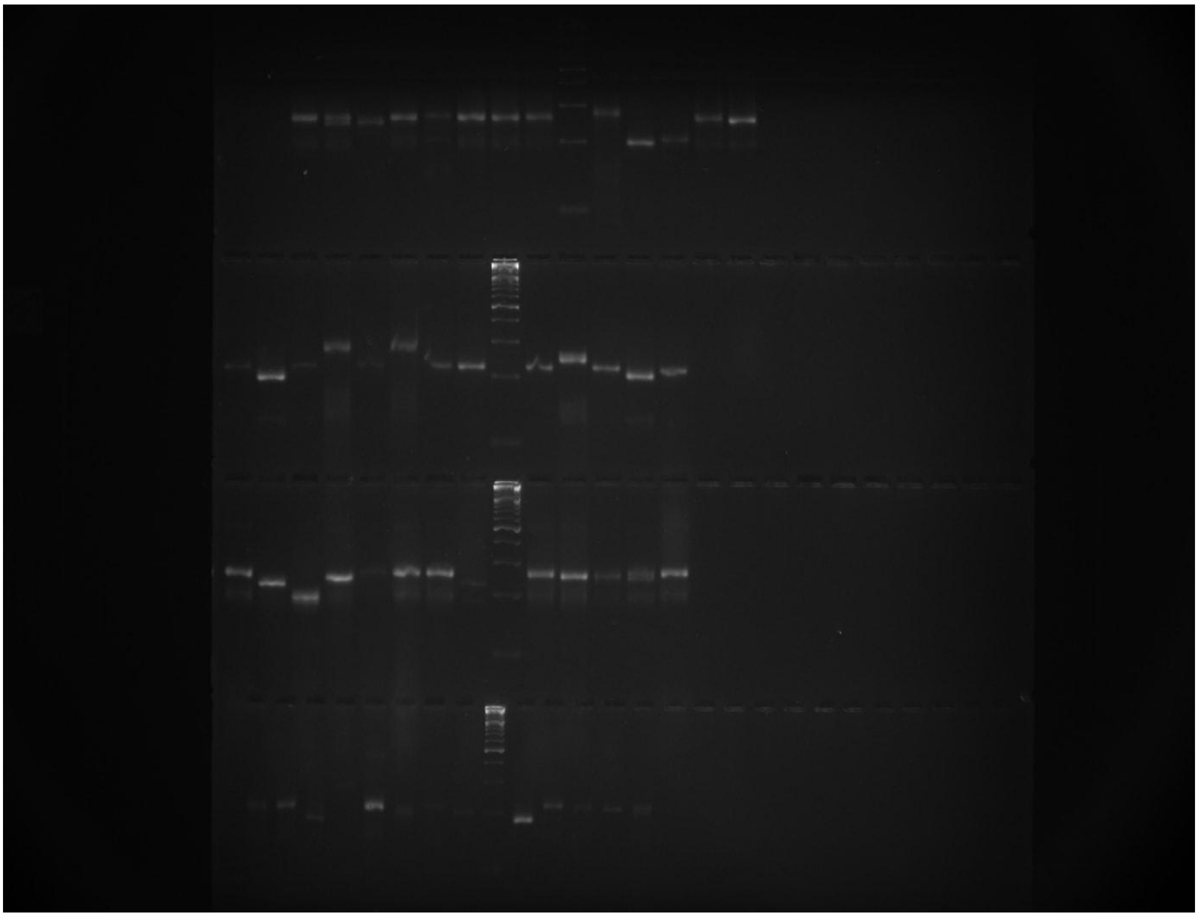

Supplementary Figure VI. The full length gel profiles of markers generated using SSR primers MB-13, VU-23, VU-25 and MB-36 respectively in control and M_4 high yielding mutants of cowpea varieties Gomati VU-89 and Pusa-578.

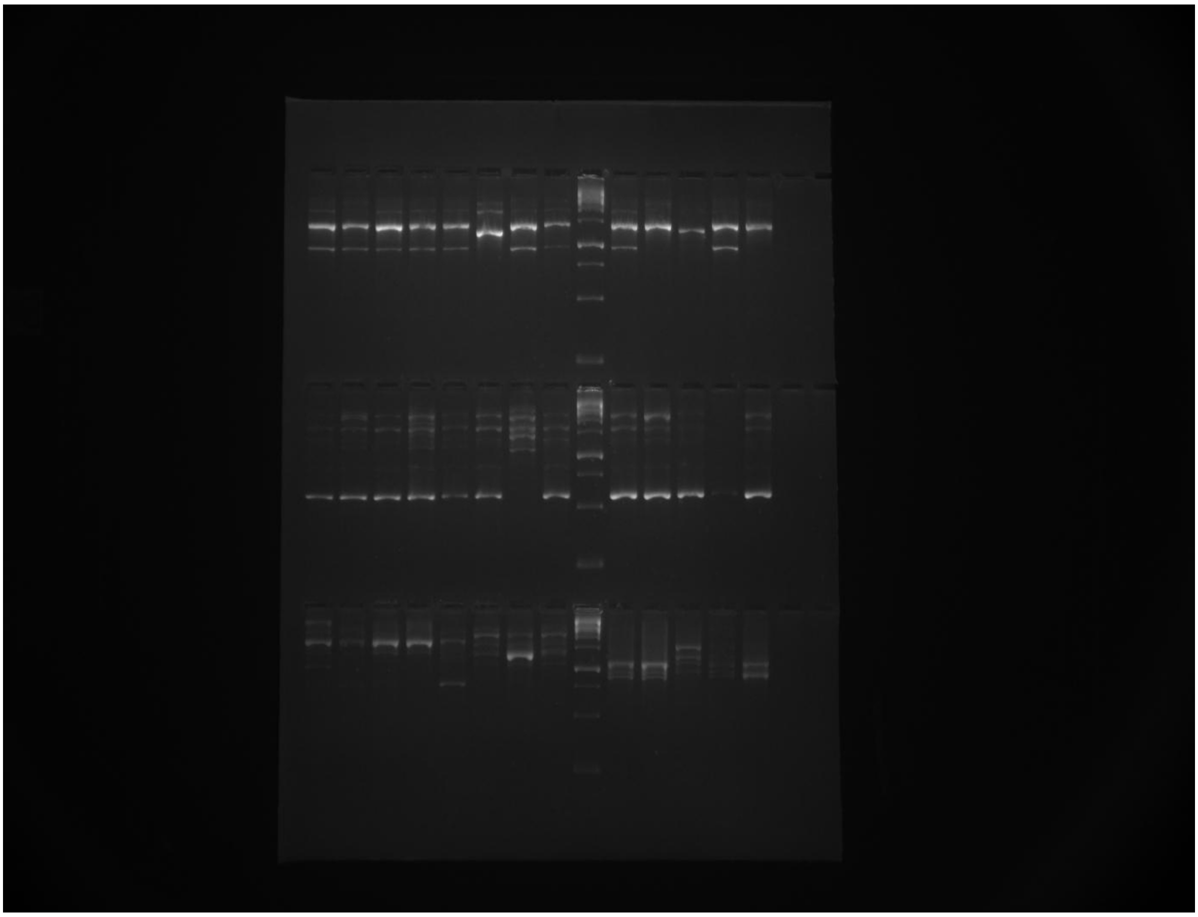

Supplementary Figure VII. The full length gel profiles of markers generated using CAAT primers CBDP-17, CBDP-19 and CBDP-16 respectively in control and M_4 high yielding mutants of cowpea varieties Gomati VU-89 and Pusa-578.

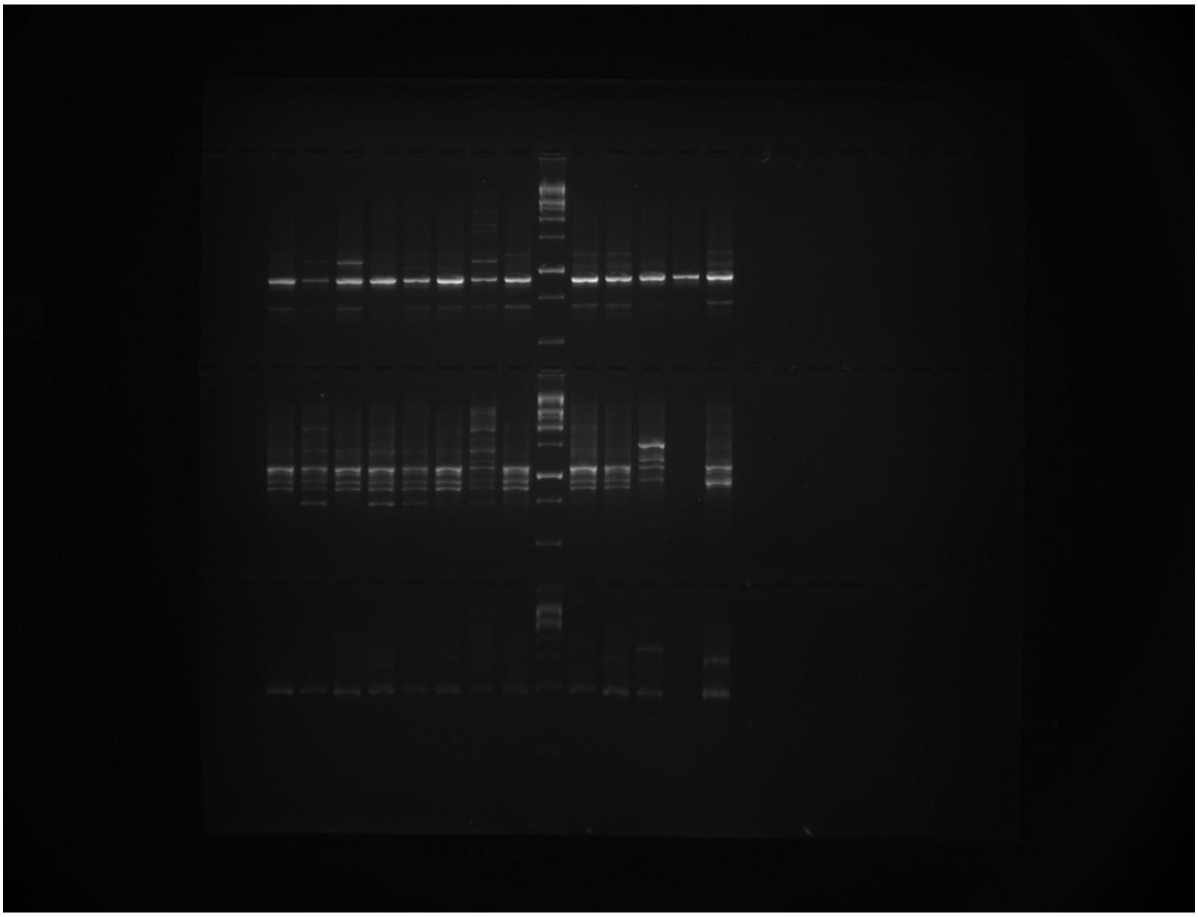

Supplementary Figure VIII. The full length gel profiles of markers generated using CAAT primers CBDP-12, CBDP-10 and CBDP-25 respectively in control and M_4 high yielding mutants of cowpea varieties Gomati VU-89 and Pusa-578.

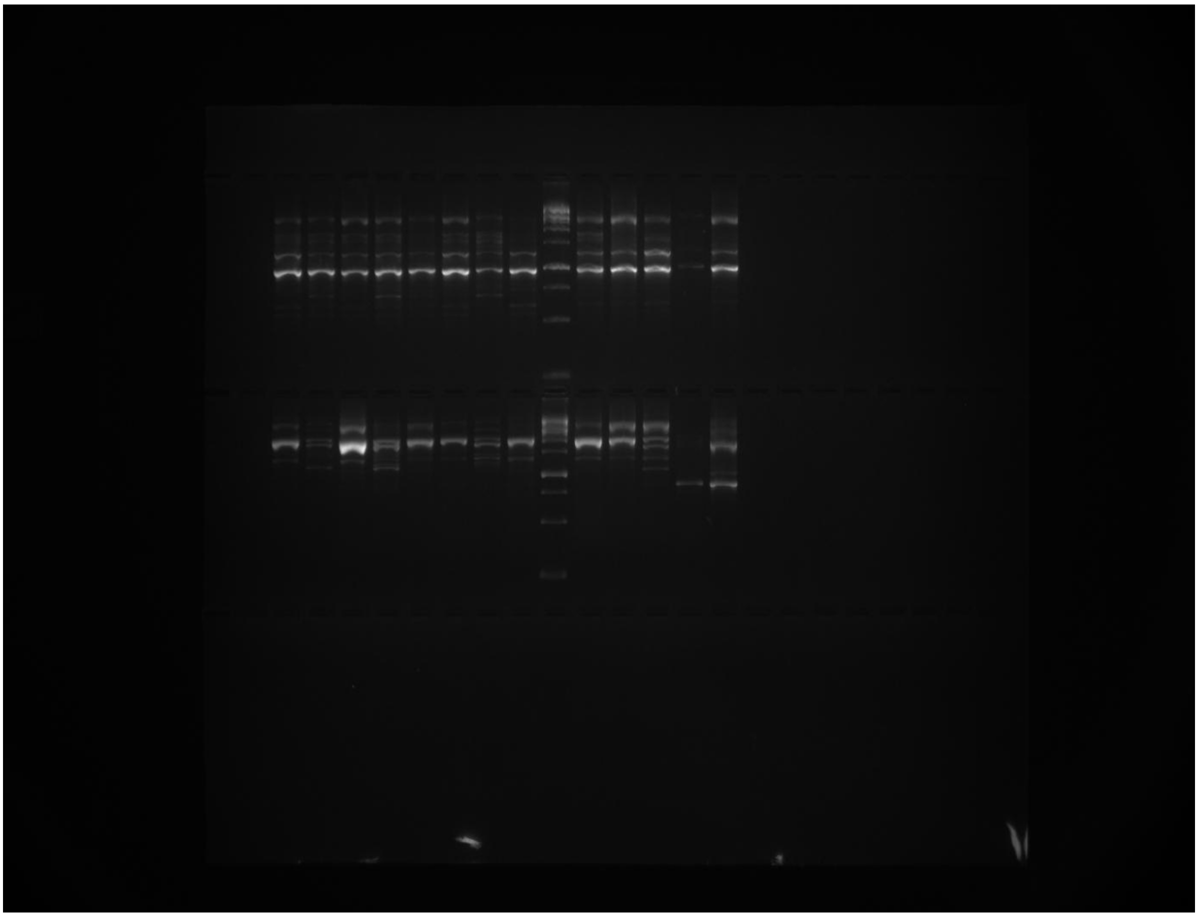

Supplementary Figure IX. The full length gel profiles of markers generated using CAAT primers CBDP-13, and CBDP-8 respectively in control and M_4 high yielding mutants of cowpea varieties Gomati VU-89 and Pusa-578.

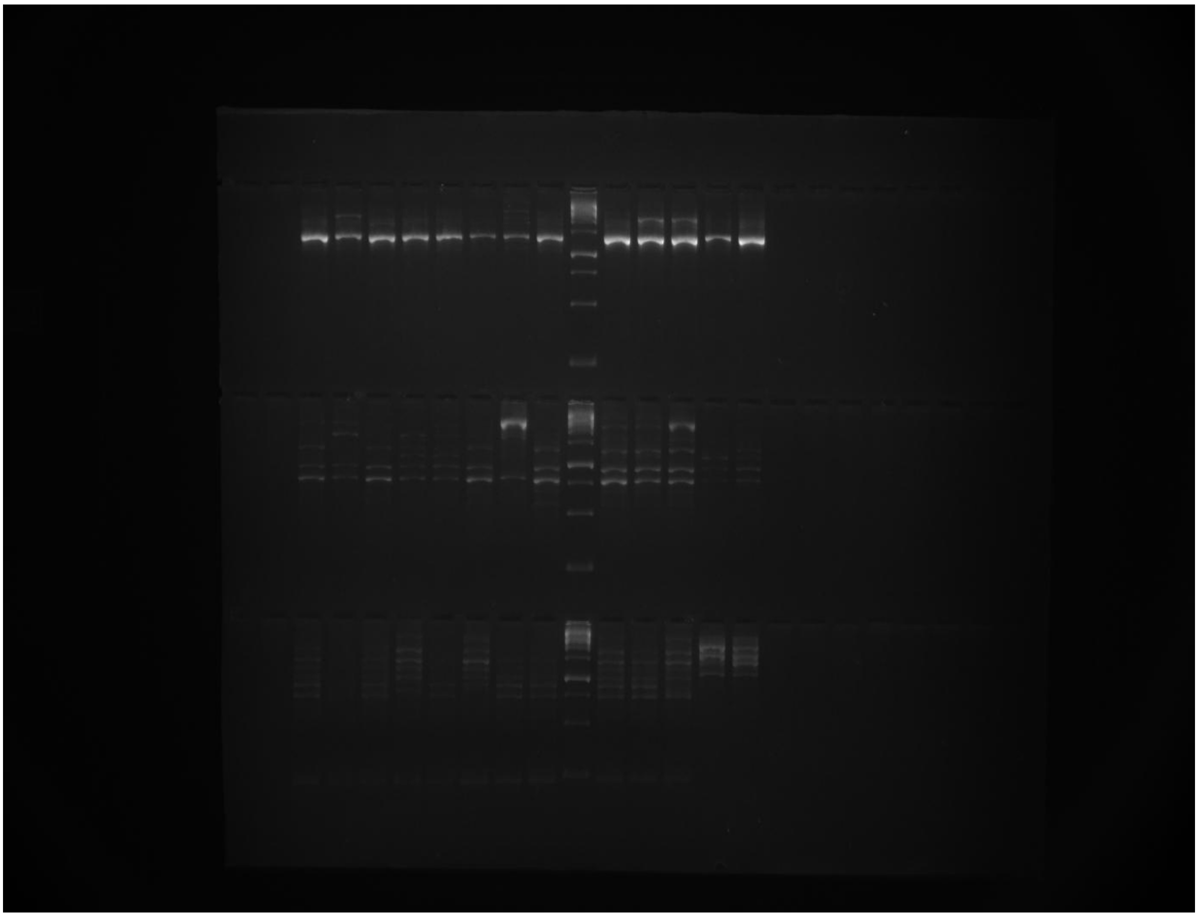

Supplementary Figure X. The full length gel profiles of markers generated using CAAT primers CBDP-18, CBDP-20 and CBDP-7 respectively in control and M_4 high yielding mutants of cowpea varieties Gomati VU-89 and Pusa-578.

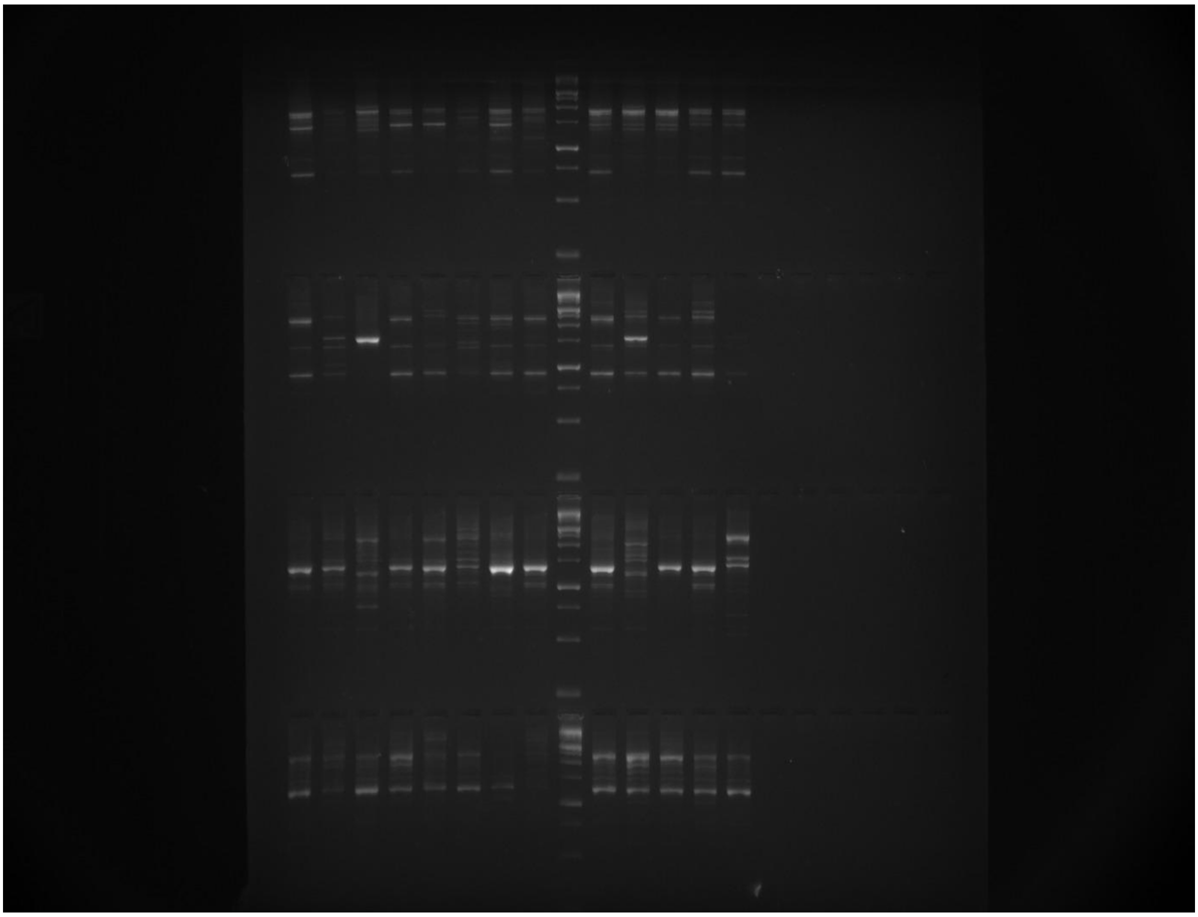

Supplementary Figure XI. The full length gel profiles of markers generated using CAAT primers CBDP-1, CBDP-3 and CBDP-6 respectively in control and M_4 high yielding mutants of cowpea varieties Gomati VU-89 and Pusa-578.
